# An image-computable spatio-chromatic receptive field model of the midget retinal ganglion cell mosaic across the retina

**DOI:** 10.1007/s10827-026-00930-z

**Published:** 2026-04-01

**Authors:** Nicolas P. Cottaris, Brian A. Wandell, David H. Brainard

**Affiliations:** 1 Department of Psychology, University of Pennsylvania, Philadelphia, PA USA; 2 Department of Psychology, Stanford University, Stanford, CA USA

**Keywords:** Retinal ganglion cells, Receptive field, Model

## Abstract

Image-computable models of primate retinal ganglion cell (RGC) mosaics that are synthesized and constrained jointly by optical, anatomical and physiological properties, and which operate on images defined by their spatial-spectral radiance, do not currently exist. Here, we deploy a novel computational framework which synthesizes mosaics of linear spatio-chromatic receptive fields (RFs) of ON midget RGCs (mRGCs) by integrating published anatomical, physiological, and optical quality measurements, all varying with eccentricity. We use the synthesized mRGC mosaics to simulate both *in vivo* and *in vitro* physiological experiments and demonstrate the model’s consistency with published data. The model enables computation of how visual performance is shaped by the representation of visual information provided by the linear spatiochromatic processing stage of midget RGCs. The developed computational framework carefully accounts for the effect of physiological optics on mRGC responses, enables comparison of *in vivo* and *in vitro* data, and allows exploration of how different assumptions about RF organization, such as selectivity for the type of cones pooled by the RF center mechanism, affect physiological responses and psychophysical performance. The open-source and freely available implementation provides a platform for understanding how the linear spatiochromatic receptive field representation of the mRGCs shapes visual performance, as well as a foundation for future work that incorporates response nonlinearities, temporal filtering, and extends to additional RGC mosaics.

## Introduction

An important aim in computational visual neuroscience is to create accurate computer simulations of how neurons in the visual pathways encode and respond to visual scenes. These simulations, often called digital twins, are a quantitative description of the visual system. They enable links between the neural representation and perception and provide a tool for evaluating the effects of blinding disease and its treatment.

Over the last ten years we have built an open-source software platform, ISETBio (Image Systems Engineering Tools for Biology) (Wandell et al., 2022), which serves as a digital twin for the initial stages of the human visual system. Previously, we described how ISETBio models (a) the formation of the retinal image, (b) the excitation of the cone photoreceptors, (c) phototransduction, and (d) fixational eye movements (Cottaris et al., 2019, 2020; Lian et al., 2019). We and others have employed ISETBio to model human vision, including sensitivity to spatial contrast (Cottaris et al., 2019, 2020), the impact of chromatic aberration on acuity (Nankivil et al., 2024), the encoding of information from natural images captured by cones (Zhang et al., 2022), the effects of optics and cone density across the visual field on performance (Kupers et al., 2019), and the influence of initial visual signals on tasks like judging surface properties and lighting (Ding et al., 2019; Singh et al., 2018). We also used ISETBio to help interpret experimental measurements of retinal ganglion cells (Godat et al., 2022).

Here, we describe an extension of ISETBio which models the mosaic of a class of retinal ganglion cells (RGCs), the midget RGC (mRGC) mosaic. RGCs are the only pathway for information transmission from the retina to the brain, and their properties surely impact visual performance on many tasks. The spike trains transmitted via the axons of one million RGCs that form the human optic nerve, represent the signals from roughly 6.5 million cones and 110 million rods (Osterberg & Andersen, 1935; Polyak, 1941). Of these RGCs, mRGCs are a particularly important subtype, comprising 80% of the perifoveal RGCs and 45% of the peripheral RGCs. In the very central fovea, it has been estimated that the mRGCs are 95% of the RGC population (Dacey, 1993).

The role of the mRGCs in limiting spatial and color vision is still debated (Patterson et al., 2019). Simulation of performance using image computable models of the mRGC mosaic offers a powerful tool for understanding the visual information encoded by these cells, especially because they are very hard to measure and isolate experimentally. We have four primary goals for this human retina model.

First, the model must distinguish the contributions of the eye’s optics and photoreceptors from the subsequent post-receptoral retinal circuitry. This separation is crucial for incorporating key physiological measurements, some of which are made *in vitro* without the eye’s optics. Failing to isolate the optical effects would prevent us from using this vital collection of data.

Second, the model must capture responses across a large portion of central retina. This is important because we and others are interested in how the retinal representation shapes performance not just in the fovea but also for peripheral viewing.

Third, the model must integrate diverse data types, including optical, anatomical, and physiological measurements. A comprehensive formulation is necessary because retinal ganglion cell (RCG) responses are shaped by all three of these factors.

Fourth, we aim for an extensible framework. The current implementation uses a linear spatiochromatic receptive field, which serves as a good initial approximation. The framework is designed to incorporate future extensions—such as response nonlinearities, temporal dynamics, and additional RGC classes—to improve the model’s accuracy over time. The following points describe how our implementation achieves these goals.

1. *Separating representations.* Our mRGC model operates on the cone mosaic signals. This design isolates the post-receptoral circuitry (cone-to-mRGC), which is the pathway measured in *in vitro* experiments where the eye’s optics are removed (Field et al., 2010; Wool et al., 2018). This separation is also valuable for interpreting experiments that use adaptive optics to eliminate optical blur (Godat et al., 2022). While the components are separable, our implementation integrates the optics, cone sampling, and mRGC circuitry into a complete, image-computable pipeline. This full pathway allows us to simulate the transformation of a visual stimulus into an mRGC response, matching the conditions of *in vivo* measurements (Croner & Kaplan, 1995; Lee et al., 2012; Reid & Shapley, 1992) and enabling predictions of human performance under natural viewing conditions.
2. *Representation across the visual field.* Visual performance varies across the visual field, and a key contribution of our model is that it allows computation of the mRGC representation continuously across the retina from the fovea out to 30$^\circ$, along any meridian. Achieving this goal required implementation of novel algorithms for synthesizing mRGC RF mosaics.
3. *Multiple data types.* By explicitly representing different biological stages, our model enables algorithms that combine anatomical, physiological, and optical data. Incorporation of multiple types of measurements from the literature is critical because at present no one type of data sufficiently constrains mRGC properties across the visual field.
4. *Extensible.* The current implementation is a linear spatial pooling model, a useful approximation for stimuli with modest contrast. The software’s modular design provides a foundation for future extensions. We can incorporate known nonlinear properties that shape mRGC responses, including phototransduction effects (Chen et al., 2024), spatial and static nonlinearities, which often differ between ON and OFF pathways (Freeman et al., 2015; Hong & Rieke, 2024; Raghavan et al., 2023; Turner & Rieke, 2016), temporal dynamics (Benardete & Kaplan, 1997) and response noise (Croner et al., 1993). Furthermore, the mRGC model is a suitable base for developing models of other types of RGCs, such as parasol and bistratified cells (Kling et al., 2024).

### Model overview

**Fig. 1:**
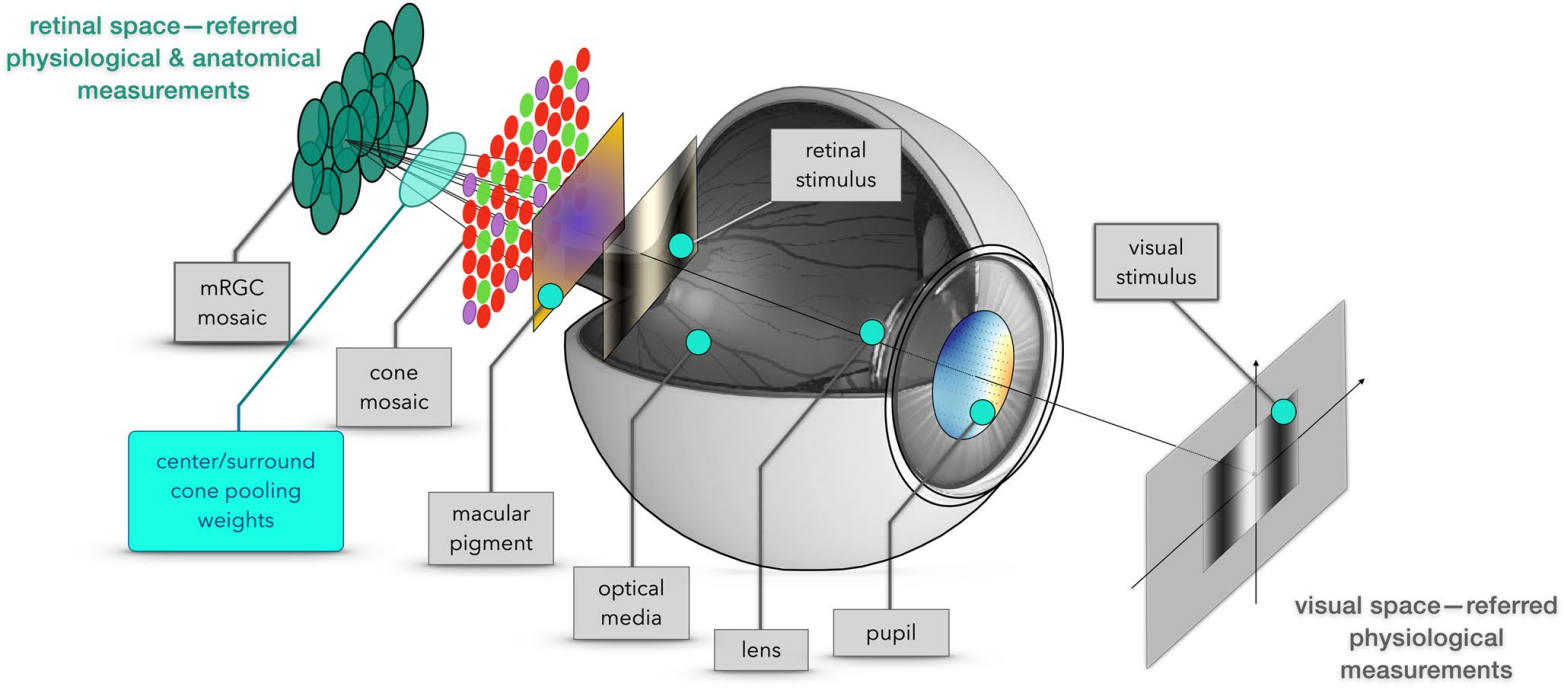
Model overview. The extant ISETBio model computes the mosaic of cone excitations. The model mRGCs are obtained by connecting their RF center and surround subregions to the cone mosaic. The connectivity matrix is constrained by anatomy and optimized through forward simulation of physiological measurements, so that the synthetic mRGCs are consistent with optical, anatomical and physiological data across the visual field

Figure 1 provides a model overview. Computation begins with the image spatial-spectral radiance, such as produced by a calibrated monitor. A model of the human optics, including chromatic aberrations and spectral filtering by the lens, is used to compute the retinal irradiance. Retinal irradiance is spectrally filtered by the macular pigment and then spatially and spectrally sampled by the cone photoreceptor mosaic. The parameters of the optics, macular pigment and cone mosaic all vary across the visual field, according to measurements in the literature (Cottaris et al., 2019).

The mRGC mosaic extension is composed of spatial receptive fields (RFs) whose center and surround responses are weighted sums of signals from the cone mosaic. The wiring between the input cone mosaic and the mRGC mosaic is initially determined based on anatomical constraints, such as cone and mRGC densities, and is subsequently refined using optimization algorithms that align the model’s spatial RF properties with physiological measurements.

A key challenge is the scarcity of *in vitro* physiological data across the visual field which could be used to directly determine the wiring between the two mosaics. To address this, our framework primarily leverages more widely available *in vivo* data to derive the wiring, while validating the synthesized model against *in vitro* data where it exists. The resulting model is simultaneously consistent with cone light encoding, anatomical properties (including those of mRGCs and H1 horizontal cells), and both *in vitro* and *in vivo* physiological data. This makes the model versatile for simulating visual stimulation under *in vivo*, *in vitro*, and adaptive optics paradigms.

#### Relationship to previous computational models of RGCs

We are not the first to construct computational models of mammalian RGCs (Ly et al., 2025; Pillow et al., 2005; Somaratna & Freeman, 2025; Wohrer & Kornprobst, 2009). Our work complements these earlier efforts, in the sense that we extend RGC modeling in ways not captured by these models. More specifically, to our knowledge, no previous image-computable model of RGCs has attempted to realistically capture the effects of the front end encoding in the visual system, particularly the eccentricity and wavelength-varying nature of physiological optics, and the eccentricity-varying spatio-chromatic properties of the cone mosaic. Instead previous models of RGCs have either not incorporated the optics (Ly et al., 2025; Wohrer & Kornprobst, 2009), or employed simplified optical models (Somaratna & Freeman, 2025). Similarly, previous RGC models have either not incorporated the cone mosaic (Wohrer & Kornprobst, 2009), or employed simplified models of the cone mosaic (Ly et al., 2025; Somaratna & Freeman, 2025). Finally, previous models operate on stimuli specified by achromatic light intensity rather than spectral radiance (Ly et al., 2025; Somaratna & Freeman, 2025; Wohrer & Kornprobst, 2009). As such, previous models do not capture the rich spatio-chromatic interactions between stimuli and physiological optics and how their combined effects shape RGC responses. Indeed, we have recently shown that the spatio-chromatic interactions between stimuli and physiological optics can have profound effects of the response properties of midget ganglion cells (Cottaris et al., 2024).

On the other hand, previous computational models of RGCs have focused on other important components of the RGC circuit that our linear spatio-chromatic model does not address. These include processing by retinal interneurons (Hennig et al., 2002; Ly et al., 2025; Somaratna & Freeman, 2025; Wohrer & Kornprobst, 2009), temporal dynamics (Hennig et al., 2002; Ly et al., 2025; Somaratna & Freeman, 2025; Wohrer & Kornprobst, 2009), contrast grain control (Pillow et al., 2005; Wohrer & Kornprobst, 2009), and spike generation (Ly et al., 2025; Pillow et al., 2005; Wohrer & Kornprobst, 2009). These are directions that could be profitably incorporated into our modeling work, as outlined in section 4.2.2.

#### Paper organization

The remainder of this paper is organized as follows.

- In section 2 we describe the model’s construction stages, including, how the mRGC receptive field lattice is generated from anatomical data (section 2.1), how cones get connected to the mRGC RF centers using anatomical and physiological constraints (section 2.2), and how cone connections to mRGC RF surrounds are derived by optimizing against *in vivo* data (section 2.3).
- In section 3 we present, validate, and discuss first applications of the model. Specifically, we illustrate examples of synthesized mRGC RFs (section 3.1), confirm that the model mRGC spatial RFs are consistent with *in vivo* data (section 3.2), and *in vitro* data (section 3.3), demonstrate the significant impact of physiological optics (section 3.4), and how simpler Difference-of-Gaussians models can fail to capture the true surround pooling (section 3.5). Finally we illustrate how the model can be used to estimate the contribution of the mRGC mosaic to spatiochromatic contrast sensitivity across the visual field (section 3.6).
- In section 4, we summarize our work, discuss ongoing applications of the model in its current stage, and discuss the model’s present limitations and planned expansions.

## Methods

The synthesis of mRGC RF mosaics occurs in three stages. In the first stage, we generate spatial lattices representing the RF centers of cells in the mRGC mosaic and the position of cones in the cone mosaic that provides the input to the mRGC mosaic. In the second stage, we connect the input cone mosaic to the RF centers of cells in the mRGC mosaic. In the third stage, we connect the input cone mosaic to the RF surrounds of cells in the mRGC mosaic.

### Generating the spatial position lattice of mRGC RF centers (Stage 1)

**Fig. 2:**
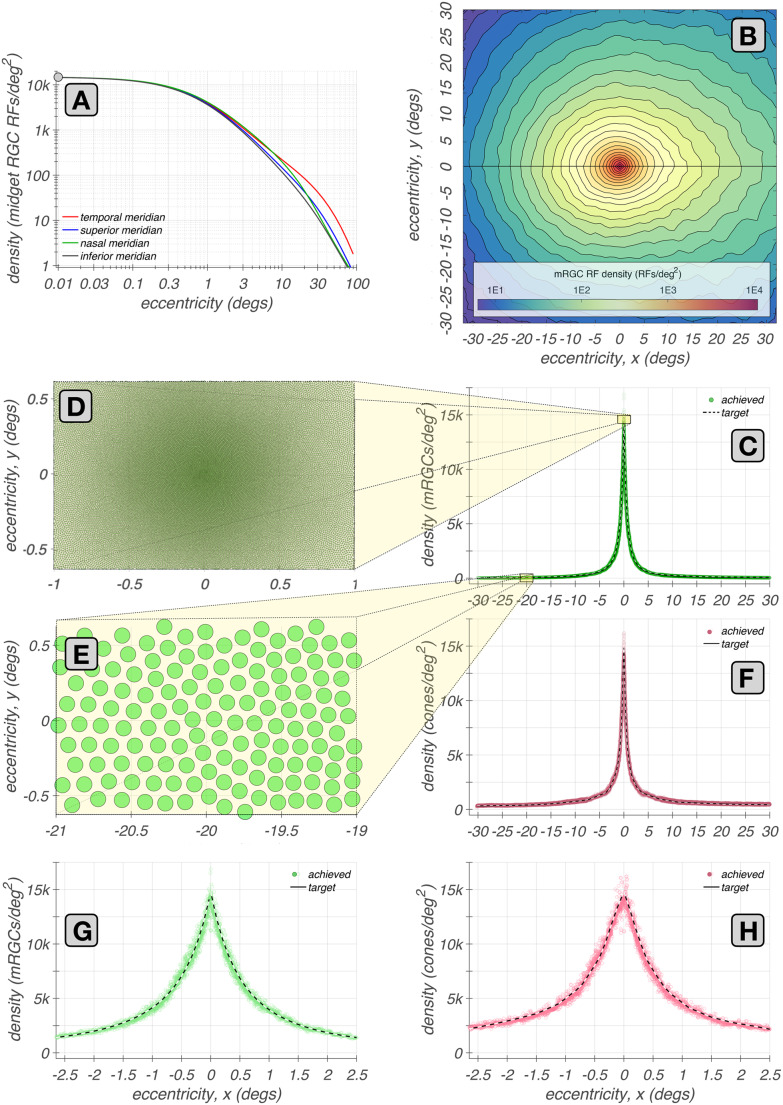
Eccentricity-varying mRGC RF position lattices. **A:** Meridian density functions of mRGC RFs (Watson, 2014). **B:** Two-dimensional mRGC RF density map obtained by interpolating the four meridian density functions. **C:** Achieved and target densities of mRGC RF centers along the horizontal meridian (green disks and white dashed line, respectively). **D & E:** Examples of $2^{\circ } \times 1^{\circ }$ lattices of mRGC RF centers at eccentricities of 0$^\circ$ and 20$^\circ$ along the temporal meridian, respectively. **F:** Achieved and target densities of cones along the horizontal meridian (maroon disks and white dashed line, respectively). **G & H:** Achieved and target mRGC and cone densities within the central 5$^o$

We begin by generating a lattice that represents the (*x*, *y*) positions of mRGC RF centers. This process comprises three sub-stages, components of which are illustrated in Fig. 2.

- **Stage 1A:** We estimate the mRGC RF center densities along the four principal meridians (0$^\circ$, 90$^\circ$, 180$^\circ$, and 270$^\circ$). These estimates are based on human data (Curcio & Allen, 1990; Watson, 2014). We take the ON–center mRGC density to be half of the total mRGC density, ignoring the possible density differences between ON– and OFF–center mRGCs. The meridian functions are depicted in Fig. 2A.
- **Stage 1B:** We generate a continuous, two-dimensional map representing the mRGC RF density map, which is depicted in Fig. 2B. This map is created by linearly interpolating the meridian estimates, and it serves as a target for the lattice synthesis algorithm in the next stage.
- **Stage 1C:** We synthesize a sampling lattice that represents the (*x*, *y*) positions of the mRGC RF centers. The lattice is created using the iterative algorithm that we introduced in earlier work (Cottaris et al., 2019) for generating cone mosaics, replacing the two-dimensional cone density map with the target mRGC RF density map. A typical lattice of mRGC RF positions is obtained after about 1,300 iterations and has a density that varies smoothly over space, matching the target density, as illustrated in Fig. 2C & G. Example lattices of mRGC RF centers synthesized at eccentricities of 0$^{\circ }$ and 20$^{\circ }$ along the temporal horizontal meridian, are depicted in Fig. 2D & E, respectively.

The same procedure is used to generate the lattice that represents the (*x*, *y*) positions of cones, using the meridian densities of cone photoreceptors in human retina (Curcio et al., 1990) as targets. The density of cones in the synthesized cone lattice also varies smoothly over space and matches closely the target density, as illustrated in Fig. 2F & H.

### Connecting cones to mRGC RF centers (Stage 2)

The connections between cones and mRGC centers are constrained by (1) anatomical data across the retina, specifically, the ratio of densities of mRGC RF centers to cones (Watson, 2014), and (2) *in-vitro* physiological data from peripheral retina, that (a) indicate that, unlike OFF–center mRGCs, which draw indiscriminately from all three cone types (Field et al., 2010; Klug et al., 2003; Patterson et al., 2019), ON–center mRGCs draw only from L– and M–cones, and (b) quantify the degree of RF center overlap between neighboring mRGCs (Gauthier et al., 2009). The connectivity between the cone mosaic and the RF centers of the ON–center mRGC mosaic is established in 3 sub-stages, summarized here.

- **Stage 2A:** In the first substage, each L– and M–cone in the input cone mosaic gets connected to a single mRGC RF center; an mRGC RF center can receive input from more than one cone. At this substage, each connected cone has unit connection weight. S-cones are not connected because they do not contribute to ON–center mRGCs. Algorithmic details regarding this substage are provided in Supplemental Section A.1.
- **Stage 2B:** The initial cone-to-RF center connectivity often results in inhomogeneities in the composition of neighboring mRGCs RF centers. These inhomogeneities are dealt with in the second substage. Here, the cone-to-RF center connections are refined to establish a balance between the spatial compactness and the spectral purity of the mRGC RF centers, which is quantified by a single parameter, $\phi$. For the body of this work, all mRGC mosaics are generated by maximizing spatial compactness, but the option to maximize spectral purity allows testing of different scenarios where mRGC RF centers may be biased to some extent towards cone type selective pooling (Field et al., 2010; Wool et al., 2018). At this substage, cones retain their unit connection weights. Algorithmic details regarding this substage are provided in Supplemental Section A.2.
- **Stage 2C:** In the third substage, the mutual exclusivity constraint enforced in substages 2A and 2B is lifted, and single cones are permitted to connect to multiple nearby mRGC RF centers. The extent of divergence varies with retinal eccentricity, being minimal in the fovea and increasing towards the periphery to match experimental observations (Gauthier et al., 2009). This is done by varying the exponent of a supra-Gaussian distribution that describes the spatial weighting profile of cone connections to the RF centers, which at this substage, become non-binary. Algorithmic details regarding this substage are provided in Supplemental Section A.3.

We illustrate Stage 2 by examining key properties of synthesized mRGC RF center mosaics at each of the three substages.

#### Mosaics with convergent-only cone connections (stage 2A)

**Fig. 3:**
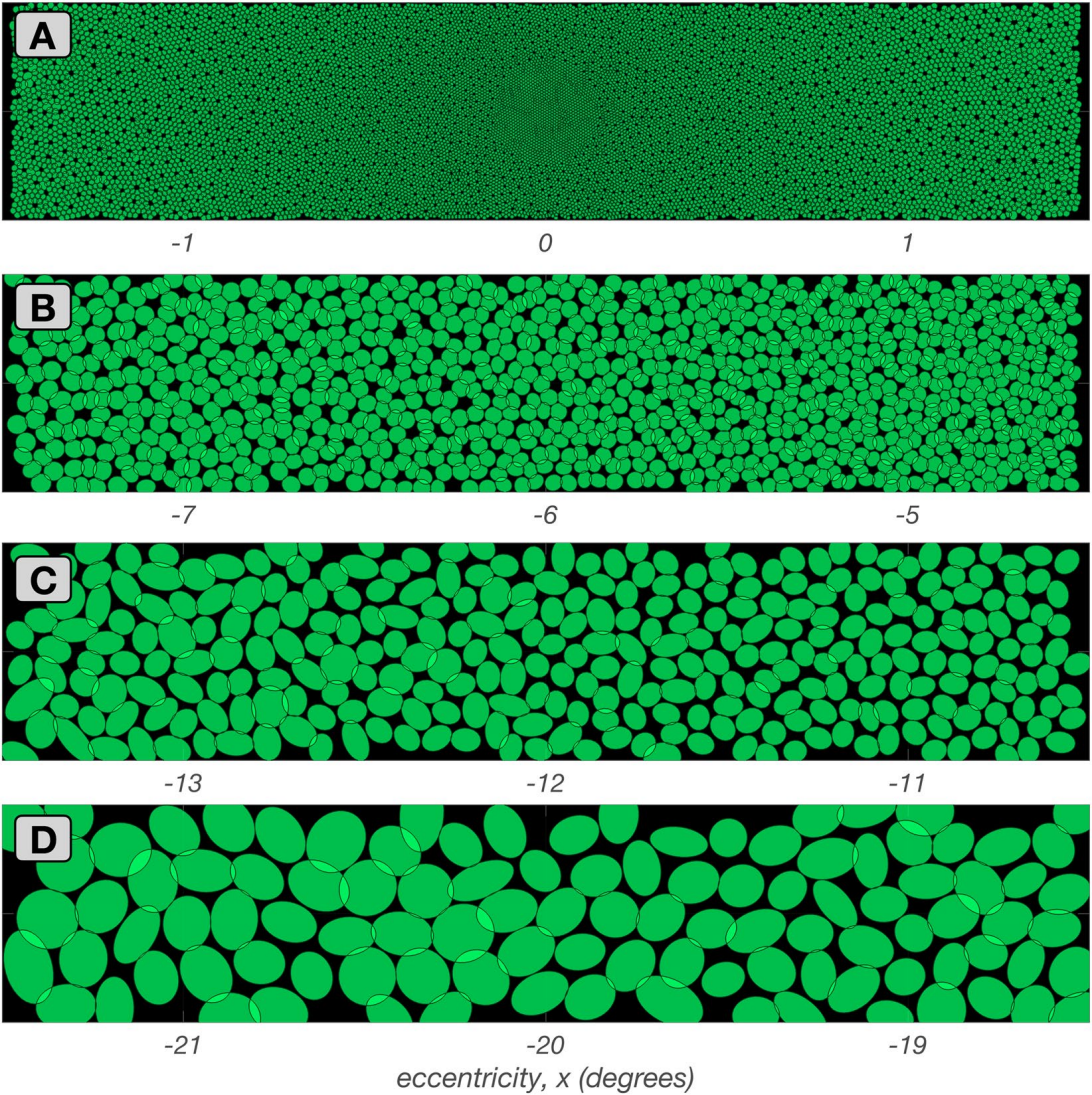
Mosaics of mRGC RF centers at stage 2A. Each panel shows a $3.0^o \times 0.5^o$ mosaic of synthesized mRGC RF centers at a different visual field location from fovea to periphery. The green ellipses depict the spatial region that encompasses all cones pooled by single RF centers. **A:** Foveal mosaic, in which RF centers receive signals from a single L– or M–cone. **B:** Mosaic centered at $6.0^o$ along the temporal horizontal meridian, in which RF centers receive signals from 2–3 L/M–cones. **C:** Mosaic centered at $12.0^o$ along the temporal horizontal meridian, in which RF centers receive signals from 3–4 L/M–cones. **D:** Mosaic centered at $20.0^o$ along the temporal horizontal meridian, in which RF centers receive signals from 6–9 cones

Example mosaics of RF centers synthesized at four eccentricities along the temporal horizontal meridian at the end of this substage are depicted in Fig. 3, where each green ellipse represents the spatial extent of the RF center of a single mRGC. At this stage, the pooling weight of each cone is unit.

For the foveal mosaic depicted in Fig. 3A, RF centers connect to just a single cone. Note how RF center sizes increase as we move towards parafoveal regions to the left and right sides of Fig. 3A. This is due to the continuously increasing, with eccentricity, cone aperture in the input cone mosaic. The empty regions in this foveal mRGC RF center mosaic correspond to the location of S-cones which are not pooled by the model.

In the parafoveal mosaic depicted in Fig. 3B, RF centers mostly receive inputs from two cones, whereas in the more peripheral mosaics depicted in Fig. 3C & D, RF centers connect to multiple cones. Note that the number of cones connecting to RF centers does not correspond precisely to RF center size, because cone aperture and inter-cone spacing both increase with eccentricity. At all eccentricities, however, mRGC RF center mosaics tile the retinal space with no spatial overlap or voids, except at the sparse positions where S–cones are located.

**Fig. 4:**
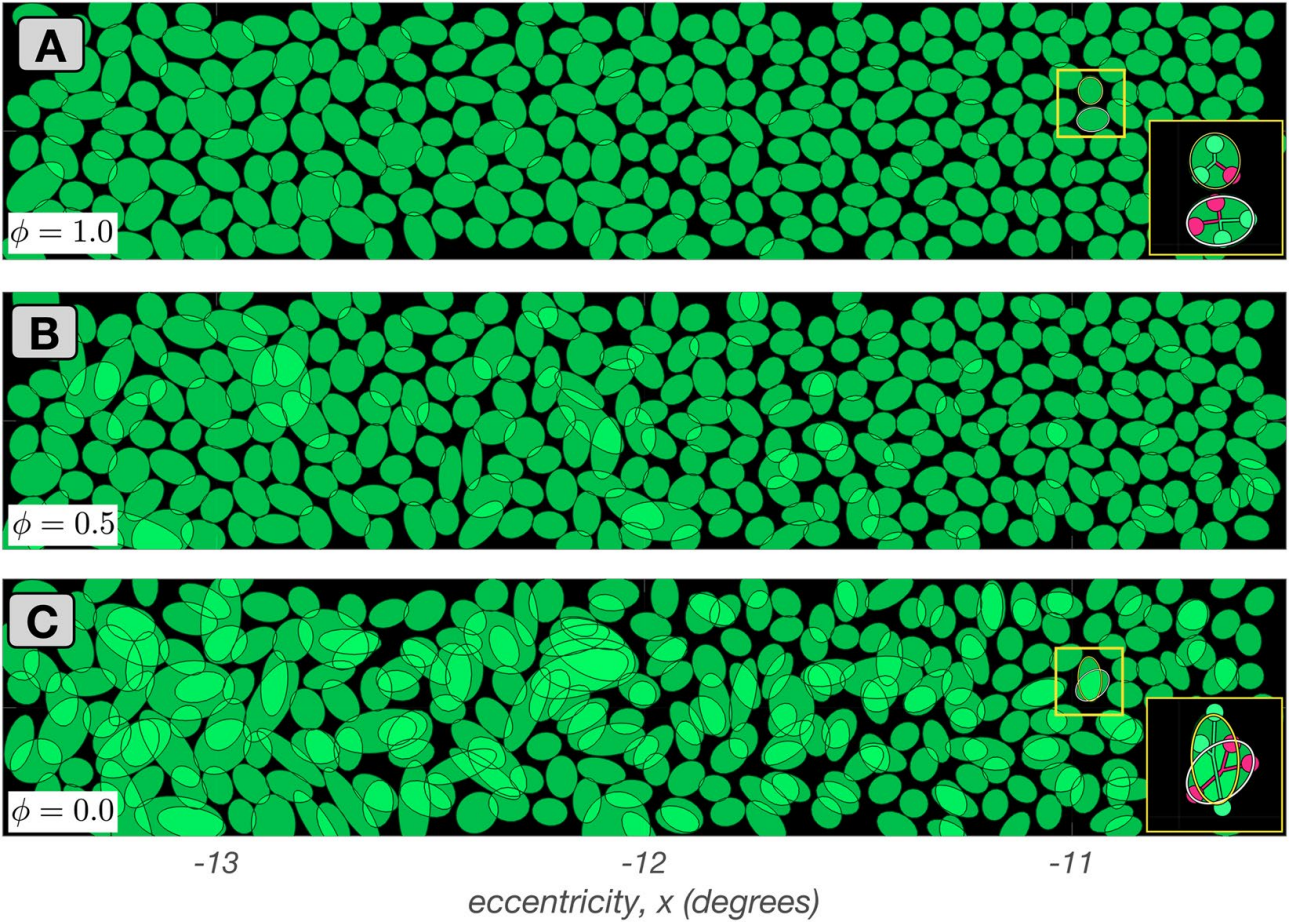
Mosaics of mRGF RF centers at stage 2B. Depicted here are $3.0^o \times 0.5^o$ mRGC mosaics, each centered at 12$^o$ along the temporal horizontal meridian, but synthesized under different values of tradeoff between spatial compactness and spectral purity, $\phi$. **A:** $\phi = 1.0$ (maximal spatial compactness). **B:** $\phi = 0.5$. **C:** $\phi = 0$ (maximal spectral purity). Insets in A and C depict pooling of cones within the RF centers of the two neighboring mRGCs contained within the yellow square. The inset in C illustrates how RF center overlap and spatial disorder is introduced as the algorithm avoids cones of different types that are close to the RF center in order to maximize the spectral purity of RF centers

#### Mosaics synthesized under different spatial compactness/spectral purity tradeoffs (stage 2B)

This substage allows for different optimizations of cone pooling within the mRGC RF centers, which is controlled by the spatial compactness/spectral purity tradeoff parameter, $\phi$. At this stage, the pooling weight of each cone is still set to unit, independent of the value of $\phi$.

Figure 4 depicts examples of mRGC RF center mosaics all synthesized at 12$^\circ$ along the temporal meridian, but under different values of $\phi$. Figure 4A depicts the mosaic synthesized under $\phi = 1$, where spatial compactness is maximal and spectral purity constraint is not enforced. Note that the RF centers tile the visual field relatively uniformly with no overlap. Figure 4B and C depict mosaics synthesized as $\phi$ decreases to 0.5 and 0.0, respectively, which increasingly enforces center connections to cones of the same type. Note that this occurs at the cost of reduced spatial compactness, as is evident by the increased spatial disorder and overlap in the RF centers.

By varying $\phi$ we can examine the effect that cone-selective pooling may have on mRGC RF spatial structure, as well as on the spatio-chromatic processing in the mRGC pathway. Current electrophysiological evidence in peripheral mRGCs RF centers favors little selective cone pooling (Field et al., 2010; Kling et al., 2020; Wool et al., 2018), i.e., a $\phi$ value of $\approx 1$. However, the degree of cone type selectivity in more central locations is not known with as much certainty. For example, there is anatomical evidence that ON–center mRGCs in the fovea contact multiple ON–cone bipolars, as opposed to OFF–center mRGCs, which contact single OFF–cone bipolars (Kolb & Marshak, 2003), and also electrophysiological evidence that the RF centers of parafoveal mRGCs appear to be pooling from more than one cones (McMahon et al., 2000). In general, the question of whether foveal mRGCs that pool from more than one cone in their RF centers are doing so selectively remains unanswered. Our modeling approach allows exploration of the benefits and tradeoffs of cone-selective pooling at any retinal eccentricity, although we do not pursue such exploration in this paper.

#### Mosaics with divergent cone connections (stage 2C)

**Fig. 5:**
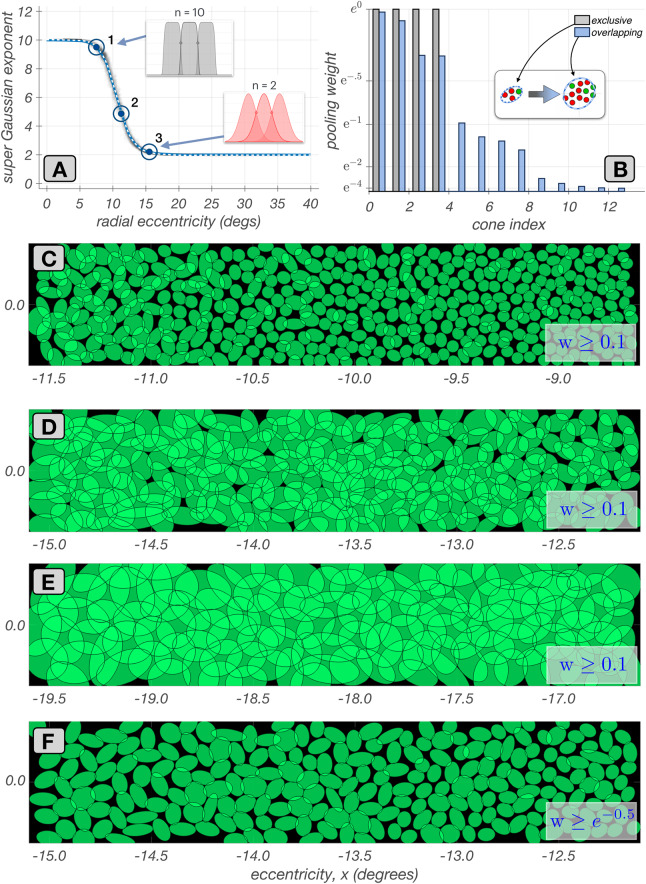
Mosaics of mRGC RF centers with divergent cone connections (stage 2C). **A:** Variation with eccentricity of the exponent of the supra-Gaussian distribution of cone pooling weights in mRGC RF centers. The exponent is set to 10 in the central retina, resulting in flat top weight distributions with zero overlap (gray histograms). As eccentricity is increased, the exponent is gradually decreased, achieving a value of 2.0, at around 15$^\circ$ (red histograms). **B:** Transformation of cone pooling weights, from binary, in mutually exclusive connections, (gray histogram) to non-binary in shared cone connections, (blue histogram) due to the supra-Gaussian distribution for an example mRGC. Insets depict the spatial arrangement of cones that are connected with binary and non-binary weights. **C, D & E:** Mosaics at 10$^\circ$, 13$^\circ$, and 18$^\circ$, respectively, along the temporal horizontal meridian with divergent cone connections. The RF center ellipses encompass the ensemble of cones with pooling weights $\ge$ 0.1. **F:** Same mosaic as **C**, but with ellipses showing cones with pooling weights $\ge e^{-0.5}$

In the final substage of establishing the wiring between mRGC RF centers and the input cone mosaic, the mutual exclusivity constraint is lifted and single cones are permitted to connect to multiple nearby mRGC RF centers. This divergence of cone connections is enabled by replacing the binary distribution of cone pooling weights in the mRGC RF centers with a supra Gaussian distribution, as illustrated in Fig. 5.

Figure 5A depicts how a progressively increasing overlap in neighboring mRGC RF centers with eccentricity is accomplished by varying the exponent of the supra-Gaussian distribution. In central retina, the exponent is kept at 10, which results in a flat top distribution of weights with minimal overlap between neighboring RF centers (gray histograms in the inset of Fig. 5A). As eccentricity increases beyond 7$^\circ$, the exponent decreases and assymptotes to a value of 2 at around 15$^\circ$, which results in Gaussian distributions of weights and a significant overlap between neighboring RF centers (red histograms in the inset of Fig. 5A).

To our knowledge, there is no physiological data on the variation with eccentricity of the divergence of cone connections to nearby mRGC RF centers. We implemented the eccentricity varying exponent as a way to smoothly bridge the gap between the fovea, where midget RF centers receive a single cone input (Kolb & Marshak, 2003; McMahon et al., 2000), and data available for the periphery, where the degree of mRGC RF overlap has been characterized (Gauthier et al., 2009). The transformation of cone pooling weights from binary and mutually exclusive to graduated and shared is depicted in Fig. 5B for an mRGC located at an eccentricity of 12$^{\circ }$, with gray and blue histograms depicting the spatial distributions of cone pooling weights before and after, respectively, substage 2C.

Figure 5C–E depict mosaics with divergent connections synthesized at three eccentricities. In these mosaic depictions, each green ellipse represents the spatial extent that encompasses all cones that are pooled by the RF center of a single mRGC with weights $\ge 0.1$. For the mosaic centered at 10$^\circ$ (Fig. 5C), divergence of cone connections has just begun. The overlap in RF centers due to the divergence of connections increases as we move in eccentricity from 9$^\circ$ on the right side to 11$^\circ$, on the left side. For the mosaic centered at around 13$^\circ$ (Fig. 5D), cone divergence and RF center overlap is higher and again increases with increasing eccentricity. For the mosaic centered at around 18$^\circ$ (Fig. 5E), divergence of cone connections has assymptoted, and we have a constant RF center overlap.

Finally, Fig. 5F provides a visualization comparable to the visualization commonly reported by *in vitro* RF mapping studies (Gauthier et al., 2009). It depicts the same mosaic as Fig. 5D, but with ellipses encompassing cones that are pooled with weights $\ge e^{-1/2} \approx 0.67$. This depiction choice makes the overlap less visually salient.

### Connecting cones to mRGC RF surrounds (Stage 3)

#### Overview

In the last stage of mRGC mosaic synthesis, we derive the cone pooling weights for the mRGC RF surrounds. Since there are no clear anatomical data on surround sizes, these weights are determined using *in vivo* characterizations of macaque mRGC visual space–referred spatial transfer functions, $\textrm{vSTF}(\omega )$, i.e., the variation in response amplitude of mRGC cells as a function of stimulus spatial frequency, $\omega$. We use the characterizations of Croner and Kaplan (1995), who measured $\textrm{vSTF}(\omega )$ for populations of mRGCs across a wide range of eccentricities.

We incorporate these data into the model using numerical optimization. More specifically, we determine the cone-to-mRGC RF surround connections such that a forward simulation of the *in vivo* physiological experiments of Croner & Kaplan through the model best reproduces the experimental data. This approach allows us to use data collected through physiological optics, which blur the stimulus in an eccentricity and wavelength dependent manner, to determine the wiring of cones to mRGC RF surrounds across eccentricities.

Importantly, the optimization is achieved while adhering to the connectivity between the cone mosaic and mRGC RF centers established in stage 2. Simultaneously, the parametric form of the spatial distribution of the surround weights is constrained based on Packer & Dacey’s characterizations of the spatial RF of macaque H1 horizontal cells (Packer & Dacey, 2002), which are the main components of the linear spatial mRGC RF surrounds (Smith et al., 1992). The use of optimization around forward simulation of an experiment to integrate data from multiple non-commensurate sources is an important innovation of our RGC modeling approach. Stage 3 proceeds in three sub-stages.

- **Stage 3A:** We begin by computing the visual space–referred cone mosaic responses to stimuli used to measure $\textrm{vSTF}$s in macaque mRGCs. This is done by presenting achromatic gratings of different spatial frequencies which are delivered to the retina through human physiological optics (Polans et al., 2015). We use human optics as a proxy of how macaque optics would have blurred the stimuli employed by the *in vivo* characterizations of Croner and Kaplan (1995), which were collected with stimuli viewed through the animal’s natural optics.
- **Stage 3B:** We derive surround cone pooling functions for a subset of target synthetic mRGCs, which span the extent of the synthesized mRGC mosaic. This optimization is done so that the ensuing target cells have (a) $\textrm{vSTF}$ characteristics that are well approximated by a Difference of Gaussians (DoG) model, with (b) parameters of the DoG model reasonably matching the DoG model parameters reported by Croner & Kaplan for macaque mRGCs at corresponding eccentricities, while (c) their surround cone pooling weights maintain macaque H1-like spatial properties as characterized by Packer & Dacey.
- **Stage 3C:** We compute surround cone pooling weights for all cells in the synthesized mRGC mosaic by evaluating the derived surround cone pooling functions at the vicinity of each mRGC’s input cone mosaic and subsequently interpolating the weights computed by the different pooling functions. A small amount of jitter in the ratio of the surround to center weights is added to simulate the variance in integrated surround to center ratios seen in the macaque data.

#### Computation of visual space–referred cone mosaic responses to stimuli used to measure vSTFs in macaque mRGCs (Stage 3A)

We employ the ISETBio machinery to compute the excitation of the input cone mosaic to achromatic gratings of different spatial frequencies delivered to the retina via physiological optics. This process captures several crucial spatio-chromatic effects in the transformation of scene radiance into cone responses: spatial and chromatic filtering by physiological optics, spectral filtering by the eye’s inert pigments, and sampling by the interdigitated trichromatic cone mosaic. To mimic the phototransduction process, cone excitation responses are converted to cone modulation responses.

In these computations, we employ human physiological optics matched to the eccentricity of each synthesized mRGC, but we adjust the defocus term of the modeled optics so as to maximize the Strehl ratio. The Strehl ratio is defined as the ratio of peak sensitivity of the optical point spread function (PSF) at the wavelength of focus, here 550 nm, to the peak sensitivity of a diffraction-limited PSF. This is done as a proxy to the experimental paradigm of Croner & Kaplan, in which corrective lenses were used to maximize cell responses at high spatial frequencies (personal communication with the late Ehud Kaplan).

#### Deriving surround cone pooling functions for a subset of target synthetic mRGCs (Stage 3B)

Croner & Kaplan reported summaries of the spatial RF characteristics across populations of mRGCs by measuring their $\textrm{vSTF}$ and then fitting a DoG model to the measured $\textrm{vSTF}$. The $\textrm{DoG}$ model defined in the spatial frequency, $\omega$, domain is given by:

1 $$\begin{aligned} \textrm{DoG}(\omega ) = K_c \cdot R_c^2 \cdot \exp { [-\pi \cdot R_c \cdot \omega ]^2 } - K_s \cdot R_s^2 \cdot \exp { [-\pi \cdot R_s \cdot \omega ]^2 } \end{aligned}$$

where $K_c$ and $K_s$ are the peak sensitivities of the RF center and RF surround mechanisms, and $R_c$ and $R_s$ are the corresponding characteristic radii.

**Fig. 6:**
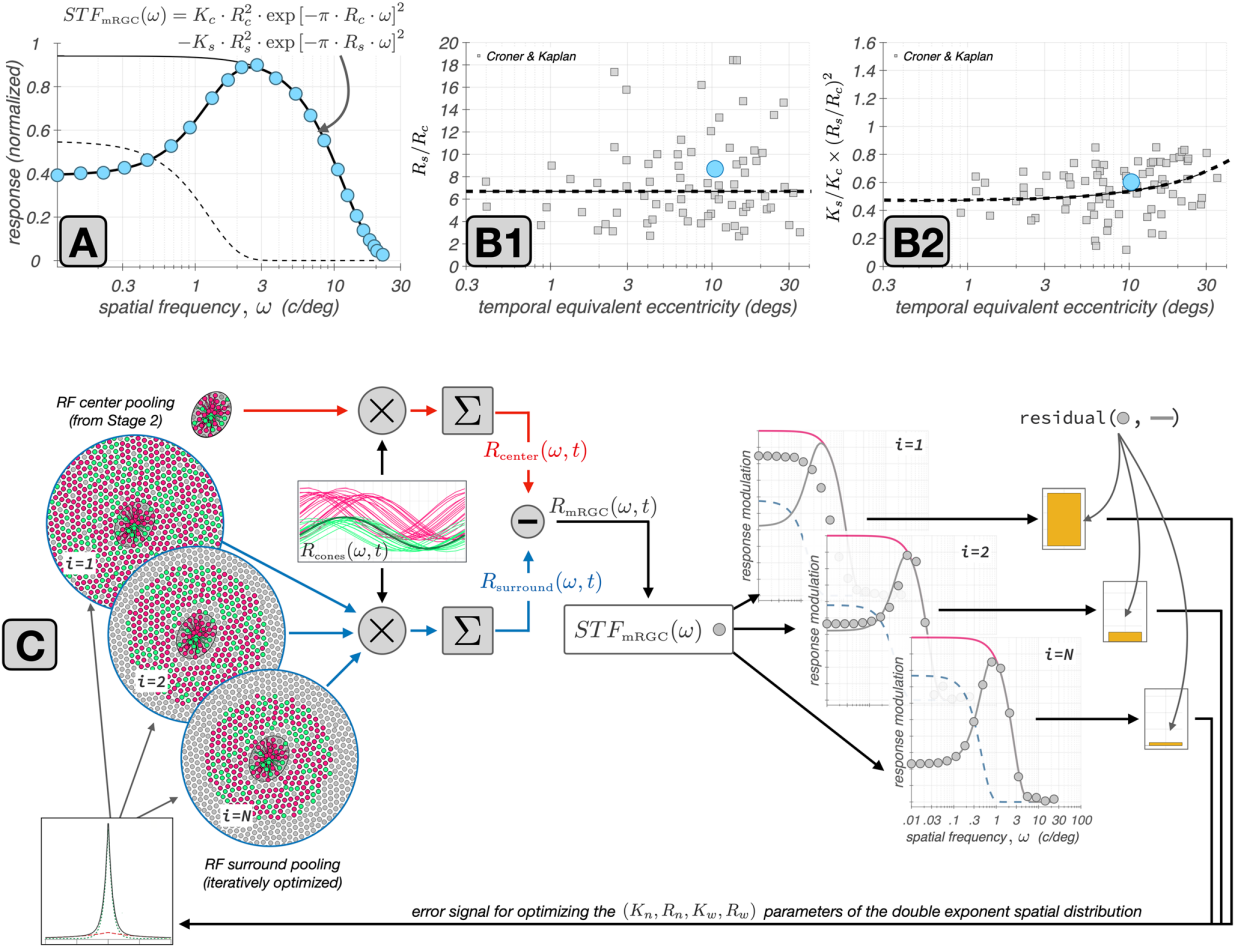
Derivation of cone weights to mRGC surrounds by forward simulation of the Croner&Kaplan vSTF measurements. **A:** Typical macaque mRGC $\textrm{vSTF}$ (cyan disks) fitted with a Difference of Gaussians model (thick black line). The model’s center and surround components are depicted by the thin black and the dashed line, respectively. **B1 & B2:** Ratios of surround to center characteristic radii, $R_s/R_c$, and ratios of surround to center integrated sensitivities, $K_s/K_c \times R_s^2/R_c^2$ as a function of eccentricity in the population of mRGCs recorded by Croner and Kaplan (1995). The dashed lines represent the trends in these two ratios as a function of eccentricity. The cyan disks depict the ratios for the example $\textrm{vSTF}$ depicted in A. **C:** Depiction of the iterative estimation of surround cone pooling weights in synthetic mRGCs by forward simulation of the Croner & Kaplan $\textrm{vSTF}$ measurements. See description in text for more details

The $\textrm{vSTF}$ of a typical macaque mRGC is depicted in Fig. 6A with cyan disks. The solid heavy line depicts the fitted DoG model, with its center and surround components depicted by the thin solid and dashed lines, respectively. The shape of the $\textrm{vSTF}$ is determined by two key measures, the ratio of surround to center characteristic radii, $R_s/R_c$, and the ratio of surround to center integrated sensitivities, $K_s/K_c \times (R_s/R_c)^2$. The distributions of these two ratios as a function of eccentricity in the population of mRGCs recorded by Croner & Kaplan are depicted by the gray squares in Fig. 6B1 & B2. The mean variation in these two ratios, shown as dashed lines, are the target values used to derive the surround cone pooling weights in the synthetic mRGCs.

The optimization process of deriving the mRGC RF surround cone pooling functions is illustrated schematically in Fig. 6C. The $\textrm{vSTF}$ of the target synthetic mRGC is computed by forward simulation of the experiment of Croner & Kaplan. The time course of responses of L– and M–cones in the input cone mosaic to a drifting grating stimulus of spatial frequency $\omega$, ${\mathrm R}_{\textrm{cones}}(\omega ,t)$, computed in Stage 3A, are depicted by the red and green traces in the rectangular panel of Fig. 6C. A spatially weighted sum of these cone responses using the RF center cone pooling weights computed in Stage 2, is used to compute the response of the RF center, ${\mathrm R}_{\textrm{center}}(\omega ,t)$. This operation, which is depicted by the red computation arm in Fig. 6C, is fixed throughout the optimization of the surround.

To compute the spatial distribution of surround cone pooling weights we impose a parametric form that is described by the sum of a narrow and a wide exponential spatial component, based on characterizations of the spatial RF properties of H1 horizontal cells by Packer and Dacey (2002). Specifically,

2 $$\begin{aligned} {\mathrm W}_s(r) = K_{\textrm{wide}} \times \exp \left[ -r/R_{\textrm{wide}} \right] + K_{\textrm{narrow}} \times \exp \left[ -r/R_{\textrm{narrow}} \right] \end{aligned}$$

where *r* is the radial distance from the RF center, $K_{\textrm{wide}}$ and $K_{\textrm{narrow}}$ are the peak sensitivities of the wide and the narrow components, respectively, and $R_{\textrm{wide}}$ and $R_{\textrm{narrow}}$ are the corresponding characteristic radii.

Beginning with a random initial value for the parameters of the double exponential distribution, we compute an initial estimate of the surround cone weights by evaluating $\textrm{W}_s(r)$ at the vicinity of the input cone mosaic that surrounds the RF center. These weights are depicted in the top-left circular panel of Fig. 6C (labeled as $i = 1$, with *i* denoting iteration). Using these initial weights we compute a weighted sum of the surround cone responses to derive the initial estimate of the surround response, $\textrm{R}_{\textrm{surround}}(\omega ,t)$ This operation is depicted by the blue computation arm in Fig. 6C.

The composite response of the synthesized mRGC is obtained by instantaneously subtracting the surround response from the center response:

3 $$\begin{aligned} {\mathrm R}_{\textrm{mRGC}}(\omega , t) = {\mathrm R}_{\textrm{center}}(\omega , t) - {\mathrm R}_{\textrm{surround}}(\omega , t) \end{aligned}$$

The amplitude modulation of ${\mathrm R}_{\textrm{mRGC}}(\omega , t)$ is taken as the value of the synthesized cell’s visual space–referred STF, $\textrm{vSTF}_{\textrm{mRGC}}(\omega )$. Repeating over a range of spatial frequencies, we obtain the initial estimate of the full $\textrm{vSTF}_{\textrm{mRGC}}$, which is depicted by the gray disks in the top-right rectangular panel of Fig. 6C, labeled as $i = 1$.

Following the experimental procedure of Croner & Kaplan, we fit the computed $\textrm{vSTF}_{\textrm{mRGC}}(\omega )$ with a DoG model. The DoG fit is depicted by the solid gray line in the top-right rectangular panel of Fig. 6C. Note that in this procedure we constrain the DoG model fit so that its shape parameters, $R_s/R_c$, and $K_s/K_c \times R_s^2/R_c^2$, both lie within a narrow range of the mean values of the $R_s/R_c$, and $K_s/K_c \times R_s^2/R_c^2$ ratios reported for macaque mRGCs at corresponding eccentricities (Croner & Kaplan, 1995). Due to this constrain, in the first iteration the residual between the computed $\textrm{vSTF}_{\textrm{mRGC}}$ and the DoG model fit to it, is large.

This residual, $||\textrm{vSTF}_{\textrm{mRGC}} - \textrm{DoG} ||$, which is depicted by the yellow bar in the right-most panel of Fig. 6C, serves as an error signal for the optimization of surround weights. The algorithm minimizes this error signal by adjusting the parameters of $\mathrm W_s(r)$, which controls the surround weights. This adjustment is also constrained, so that the parameters of $\mathrm W_s(r)$ remain within a range of the values reported in macaque H1 horizontal cells (Packer & Dacey, 2002).

When the $||\textrm{vSTF}_{\textrm{mRGC}} - \textrm{DoG} ||$ reaches a minimum value, at iteration $i = N$ in Fig. 6C, we obtain the optimized surround cone pooling function for the target synthetic mRGC. Additional details about this surround optimization method are provided in Supplemental Section B.1.

### Deriving surround cone pooling weights for each cell in the mosaic (stage 3C)

The optimization of the surround cone pooling functions is a computationaly expensive process. It is therefore conducted on a sparse spatial grid (with $N_{xy}$ nodes), which encompasses the spatial extent of the synthesized mRGC mosaic. At each node of the spatial grid, we determine the range of cone numerosities in the RF centers of nearby synthetic mRGCs, and we derive optimized surround cone pooling functions for each of the encountered RF center cone numerosities ($N_{c}$), and we do this twice, once for L-cone dominated RF centers, and once for M-cone dominated RF centers.

Once these $N_{xy} \times N_{c} \times 2$ surround cone pooling functions are derived, we compute surround cone pooling weights for all synthetic mRGCs. For each synthetic mRGC we determine the 3 nearest spatial grid nodes, and extract the optimized surround cone pooling functions that were derived at this node for the cone numerosity that matches that of the examined mRGC, for both L– and M–cone RF center dominance variants. Then we evaluate the six optimized surround pooling functions at the input cone mosaic in the vicinity of the examined mRGC, deriving six sets of surround cone pooling weights. The examined cell’s surround cone pooling weights are determined by interpolating the 6 sets of weights spatially, weighted inversely proportionally by the distance between the location of the examined mRGC and the location of the optimized model, and spectrally, weighted based on the relative L–/M–cone weight ratio in the RF center of the examined mRGC.

#### Adjusting the surround pooling variance

The final step in the generation of the mRGC RF surrounds is to apply a noisy scalar multiplier to all surround pooling weights of individual mRGCs. The value of this scalar is chosen so that the variance in the ratio of surround to center integrated sensitivities, $K_s/K_c \times (R_s/R_c)^2$, of the synthetic mRGCs matches the variance observed in the population of macaque mRGCs recorded by Croner & Kaplan at the corresponding eccentricity. The manipulation in $K_s/K_c \times (R_s/R_c)^2$ variance does not require re-computing the surround pooling functions. This is unlike manipulating the variance in the $R_s/R_c$ ratio, which requires re-computing the surround pooling functions.

### Computing mRGC responses from cone mosaic responses

A fully synthesized mRGC mosaic consists of two connectivity matrices: $\textrm{P}_{\textrm{center}}(i,k)$, determined in synthesis stage 2, and $\textrm{P}_{\textrm{surround}}(i,k)$, determined in synthesis stage 3, which capture the weights by which the RF center and the RF surround mechanisms, respectively, of the $k^{th}$ cell in the mRGC mosaic pools signals from the $i^{th}$ cone in the input cone mosaic.

Since the current version of the mRGC model does not contain a temporal component, the response of the $k^{th}$ mRGC to some stimulus at time instant, *t*, $\mathrm R_{\textrm{stim}}(k,t)$, is computed instantaneously by weighting the response of the input cone mosaic to that stimulus at time *t*, $\textrm{C}_{\textrm{stim}}(:,t)$, as follows:

4 $$\begin{aligned}{\mathrm R}_{\textrm{stim}}(k,t)&= \displaystyle \frac{1}{\displaystyle \sum _{i} \textrm{P}_{\textrm{center}}(i,k)} \times \dots \\& \Bigg(\begin{array}{l}\sum\limits_{i} \textrm{P}_{\textrm{center}}(i,k) \cdot \textrm{C}_{\textrm{stim}}\left( i, t \right) - \\\\\sum\limits_{j} \textrm{P}_{\textrm{surround}}(j,k) \cdot \textrm{C}_{\textrm{stim}} \left( j, t \right)\end{array} \Bigg)\end{aligned}$$

To mimic adaptation to the background stimulus, synthetic mRGCs typically operate on cone contrast responses, instead of cone excitation responses, so the $\textrm{C}_{\textrm{stim}}(i,t)$ term in the above equation is computed as:

5 $$\begin{aligned} \textrm{C}_{\textrm{stim}}(i,t) = \displaystyle \frac{\textrm{E}_{\textrm{stim}}(i,t) - \textrm{E}_{\textrm{bkgnd}}(i)}{\textrm{E}_{\textrm{bkgnd}}(i)} \end{aligned}$$

where $\textrm{E}_{\textrm{stim}}(i,t)$ is the excitation response of the $i^{th}$ cone to the examined stimulus at time *t*, and $\textrm{E}_{\textrm{bkgnd}}(i)$ is that cone’s excitation response to a uniform field, zero contrast stimulus, whose mean chromaticity and luminance match those of the examined stimulus.

### Equating eccentricity across human and macaque retina

We have built our model to describe human retina, but some of the fundamental physiological data available to constrain the model (Croner & Kaplan, 1995), and to validate the model (Croner & Kaplan, 1995; Field et al., 2010; Gogliettino et al., 2023), exists only for macaque monkey. To integrate and/or contrast data between human and macaque, we need to equate retinal eccentricity across the two species. We compared how measurements of cone density in the two species (Curcio et al., 1990; Packer et al., 1989) align when plotted in terms of millimeters of retina versus plotted in terms of visual angle. We observed better, although not perfect, alignment when eccentricity was specified in terms of millimeters of retina, and thus chose to align monkey to human data by equating millimeters of retina. More specifically, to determine the equivalent macaque angular eccentricity of a synthetic human RGC we first convert the angular eccentricity of the human RGC into its linear eccentricity (in retinal millimeters) using the formula derived by Watson (2014) based on the wide-angle schematic eye model of Drasdo and Fowler (1974). We then assume that macaque and human linear eccentricities are identical, and finally, convert the macaque linear eccentricity into its corresponding angular eccentricity (in degrees of visual angle), assuming a retinal magnification factor of 221 $\mathrm {\mu m/deg}$ for the macaque eye (Perry & Cowey, 1985).

## Results

A key feature of our model is its dual representation of mRGC receptive field (RF) properties, which separates neural circuitry from optical effects. The first representation, in *retinal space*, models the direct pooling of cone signals by the RF center and the RF surround. This describes the cell’s intrinsic spatio-chromatic filtering and is directly comparable to anatomical data and physiological measurements that bypass the eye’s optics (e.g., *in vitro* or adaptive optics experiments Godat et al., 2022; Tuten et al., 2018). In contrast, the second representation, in *visual space*, models the end-to-end processing of a stimulus as it passes through the eye’s optics to the mRGC mosaic. This representation is applicable to conventional *in vivo* physiology and psychophysical assessments of visual function.

The ability to go back and forth between cone and visual space is critical to understanding how retinal cone pooling interacts with physiological optics to generate the processing characteristics of cells in visual space, which is what ultimately determines natural visual performance. This ability is also critical in interpreting results from *in vivo* physiology in terms of the underlying retinal wiring (Cottaris et al., 2024), as well as to relating results obtained under adaptive optics viewing conditions to results obtained under natural viewing conditions (Godat et al., 2022).

In this section we illustrate and contrast spatial RF characteristics of synthetic mRGCs in the two representations and validate the properties of synthetic mRGCs against those of macaque mRGCs as characterized by *in vivo* and *in vitro* physiological studies.

### Spatial characteristics of synthesized mRGC receptive fields

**Fig. 7:**
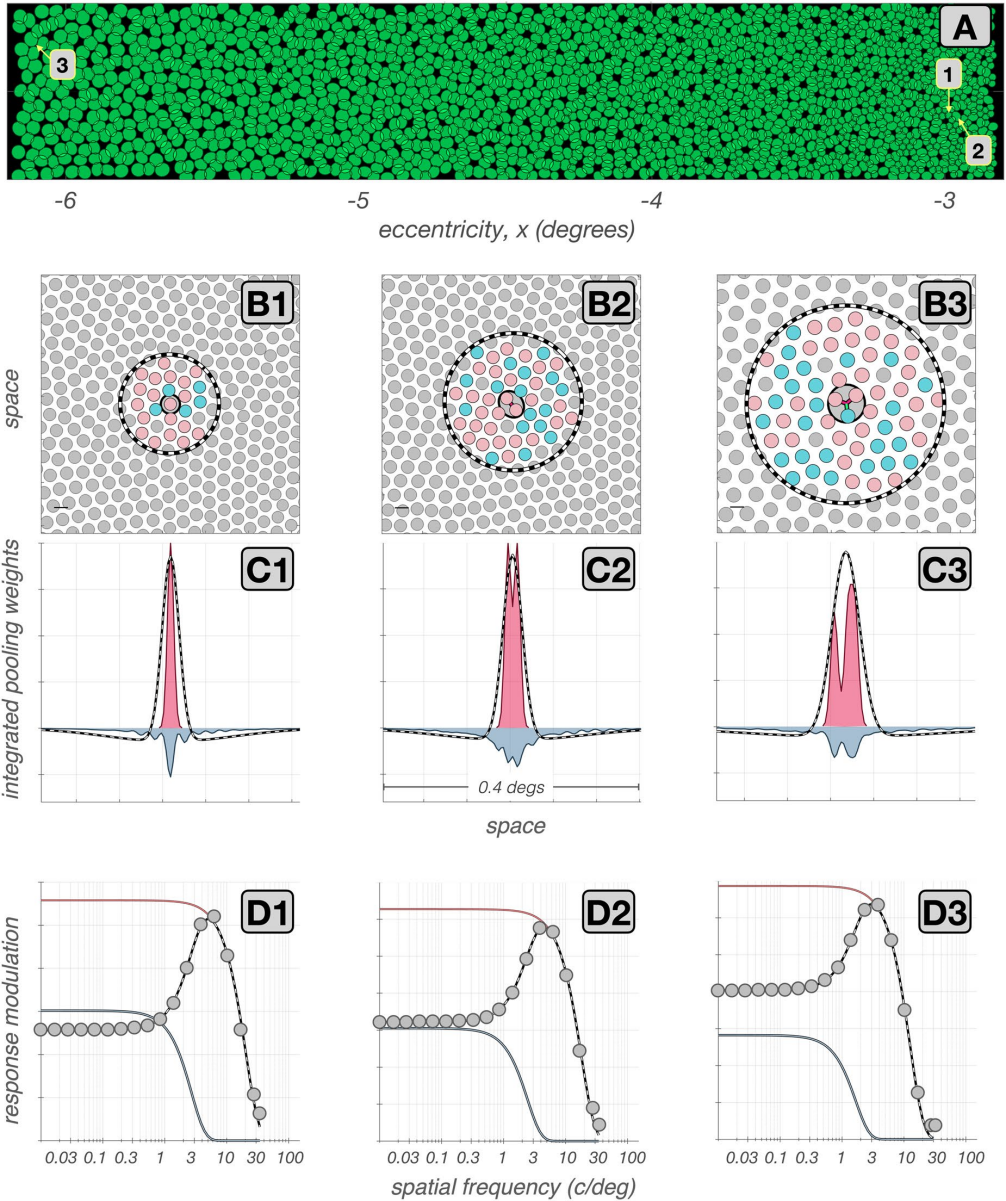
Spatial RF characteristics of synthetic mRGCs **A:** Mosaic of RF centers of an mRGC mosaic synthesized at 4.5$^\circ$ along the temporal horizontal meridian. **B1–B3:** Cone pooling maps for 3 exemplar cells whose positions within the mRGC mosaic are labeled in A. Pink and cyan disks depict L– and M–cones, respectively, with RF center pooling weights $\ge 0.1$, or with RF surround weights $\ge 0.005$. Gray disks represent either S-cones, which are not pooled in our model, or L–/M–cones with pooling weights lower than the labeling thresholds. The solid and dashed black lines depict the extents of the RF center and surround pooling regions. **C1–C3:** Y-axis integrated cone pooling weight profiles of the RF center (maroon) and the RF surround (slate) mechanisms. The dashed lines depict the visual space–referred line weighting functions as derived by fitting Difference of Gaussians (DoG) models to each cell’s $\textrm{vSTF}$. **D1–D3:** The $\textrm{vSTF}$s of the exemplar mRGCs, computed under physiological optics, are depicted by the gray disks. The gray lines depict the DoG model fits to these $\textrm{vSTF}$s, and the maroon and slate lines depict the models’ center and surround components

Spatial RF characteristics of cells in an mRGC mosaic synthesized at 4.5$^o$ along the temporal horizontal meridian are depicted in Fig. 7. Figure 7A depicts the mosaic together with numbered positions which identify the locations of three selected cells whose spatial RF characteristics are explored in detail.

Figure 7B1–B3 depict the cone pooling maps of the selected cells. Here, pink and cyan disks depict L– and M–cones, respectively, with RF center pooling weights $\ge$ 0.1, or with RF surround pooling weights $\ge$ 0.005, whereas gray disks depict cones that are either not pooled at all or pooled with a weight less than the threshold for labeling. The solid and dashed lines depict the spatial pooling extents of the RF center and surround mechanisms, respectively.

The cell depicted in Fig. 7B1 is located at an eccentricity of 3$^o$. Its RF center pools from a single L–cone and its RF surround pools from a total of 16 L– and M–cones. The cell depicted in Fig. 7B2, also located at 3$^o$, pools from two L–cones in its RF center, and its RF surround is larger, pooling from 44 L– and M–cones. The cell depicted in Fig. 7B3 is located at 6$^o$. Its RF center, which pools from 2 L–cones and 1 M–cone, and its RF surround are both larger than those of the first 2 cells. The cone pooling maps depicted in Fig. 7B1–B3 illustrate the spatial connectivity between the input cone mosaic and the center and surround subregions of mRGC RFs, but do not depict the strength of these connections. In this sense, these maps depict the type of information that is available from detailed anatomical studies.

Figure 7C1–C3 add to this view by providing information about the strength of the cone inputs in these exemplar cells. Here, the maroon and slate histograms depict the cells’ spatially integrated (along the y-axis) cone pooling weights for the RF center and the RF surround mechanisms, respectively. Note that in the cell depicted in Fig. 7C1, the double exponential spatial profile of the surround cone pooling mechanism, with a sharp peak around the RF center and more shallow weights in peripheral regions, is prominent. In the two other cells shown, this feature is less prominent.

This observation, where cells with larger RF centers have less peaked surround weights than cells with smaller RF centers is seen commonly in our synthetic mRGCs. The variation in surround pooling characteristics with RF center size results from constraints in the model, which maintain $\textrm{vSTF}$ shape parameters that are consistent *in vivo* measurements (Croner & Kaplan, 1995) while at the same time remaining consistent with the surround parametric form indicated by measurements of H1 receptive fields (Packer & Dacey, 2002).

Visual space–referred spatial transfer functions are commonly measured in *in vivo* physiological recordings that estimate spatial RF properties of mRGCs (Croner & Kaplan, 1995; Lee et al., 2012). The gray disks in Fig. 7D1–D3 depict the $\textrm{vSTF}$s of the three examined synthetic mRGCs, and the corresponding DoG model fits are depicted by the dashed gray lines. The spatial RF profiles corresponding to these DoG model fits are depicted by the dashed lines in Fig. 7C1–C3. Contrasting these inferred spatial RF profiles with the actual cone pooling profiles, it becomes evident that one cannot use characterizations obtained under physiological optics viewing conditions to directly infer the characteristics of spatial pooling of cone signals in the retina. We discuss this issue further in later sections.

### Validation against *in vivo* physiology across the visual field

To validate our model, we synthesized mRGC mosaics across a wide region of the retina, and computed $\textrm{vSTF}$s of individual mRGCs by probing them with drifting achromatic gratings of different spatial frequencies delivered to the retina under physiological optics appropriate for the eccentricity of the examined cells, simulating the experimental paradigm of Croner and Kaplan (1995). To compare synthetic and macaque mRGCs we fitted the computed synthetic cell $\textrm{vSTF}$s with the DoG model employed by Croner & Kaplan and compared the ratios of surround to center characteristic radii, $R_s/R_c$, and ratios of surround to center integrated sensitivities, $K_s/K_c \times R_s^2/R_c^2$, to those reported by Croner & Kaplan.

**Fig. 8:**
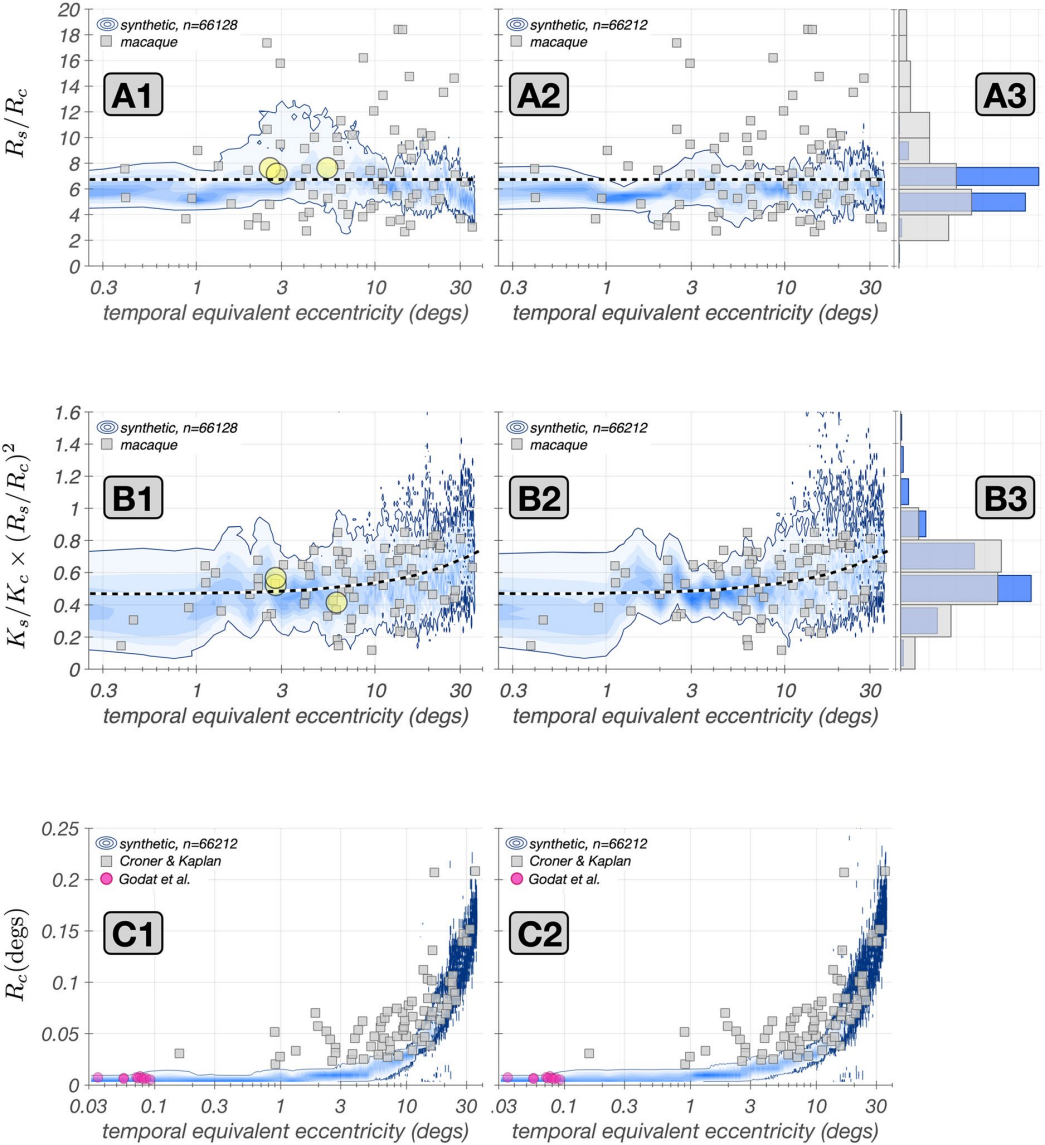
Validation against *in vivo* measurements. In all panels, gray squares depict data from the population of macaque mRGCs recorded by Croner and Kaplan (1995). Blue contours depict the probability density function of the examined parameter in a population of 66,128 synthetic mRGCs with color saturation encoding probability level. Solid blue lines represent the 5% – 95% percentile range of examined parameter. Left and right panels are for mosaics synthesized under physiological optics of two different human subjects. **A1–A2:** Correspondence in ratio of surround–to–center characteristic radii, $R_s/R_c$, across eccentricity. The dashed line represents the target value that is in effect during the optimization of the synthetic mRGC surrounds, which is the mean value of $R_s/R_c$ across the population of all mRGCs recorded by Croner & Kaplan. **A3:** Marginal histograms of $R_s/R_c$ for macaque (gray) and synthetic mRGCs (blue). **B1–B2:** Correspondence in ratio of surround–to–center integrated sensitivities, $K_s/K_c \times \left( R_s/R_c \right) ^2$, across eccentricity. The dashed line represents the target values in effect during the optimization of the synthetic mRGC surrounds, which is the trend observed with eccentricity in the population of the macaque mRGCs recorded by Croner & Kaplan. **B3:** Marginal histograms of $K_s/K_c \times \left( R_s/R_c \right) ^2$ for macaque (gray) and synthetic mRGCs (blue). **C1–C2:** Correspondence in RF center characteristic radius, $R_c$, across eccentricity. The fuschia disks represent the $R_c$ of foveolar mRGCs recorded by Godat et al. (2022), back-projected in visual space using the monkey’s own physiological optics

The results of this analysis are depicted in Fig. 8, in which the left and right panels depict data from mRGC mosaics synthesized under the physiological optics of two different human observers. Figure 8A1 and A2 compare macaque vs. synthetic mRGCs in terms of the distribution of their $R_s/R_c$ ratios. Gray squares depict the macaque mRGC data and the blue density plots depict the 5%–95% percentile range of the $R_s/R_c$ ratios in a population of 66,128 synthetic mRGCs. The three yellow disks in Fig. 8A1 correspond to the three exemplar cells illustrated in Fig. 7. Note that the $R_s/R_c$ ratios in synthetic mRGCs follow the macaque data across eccentricity for both human subjects. The synthetic data do not, however, capture the full variance seen in the macaque data, as is evident by the marginal histograms (Fig. 8A3). To capture the full variance seen in the macaque data, we could consider synthesizing multiple surround pooling functions, each with different target values of $R_s/R_c$, and then randomly selecting for each synthesized mRGC from the multiple sets.

On the other hand, the integrated sensitivity ratios, $K_s/K_c \times R_s^2/R_c^2$, of the synthetic mRGC population, depicted in Fig. 8B1–B3, capture both the trend with eccentricity and the variance of the macaque data. The variance match was achieved by enforcing a target variance in the $K_s/K_c \times R_s^2/R_c^2$ ratio of the synthetic cells as described earlier.

Note that, although we did use the mean variation with eccentricity of macaque $R_s/R_c$ and $K_s/K_c \times R_s^2/R_c^2$ ratios during construction of the model, the model was derived using additional constraints: those imposed by the densities of cones and mRGC RFs, by the spatial characteristics of H1 horizontal cells, and by the influence of human optics. These validations, therefore, check both that we have not over constrained our model in a manner that makes it inconsistent with the macaque data, and that our method of interpolating surround pooling weights from models derived at a set of discrete retinal locations works well.

We next examined the correspondence between synthetic and macaque mRGCs in terms of their visual space–referred RF center sizes, $R_c$. Recall that in synthesizing mRGC mosaics, the RF centers are constructed independently of the Croner & Kaplan physiological data, using only anatomical data and estimates of RF center overlap obtained from *in vitro* physiology in the periphery (Gauthier et al., 2009). Figure 8C1–C2 compare the distributions of $R_c$ between the macaque and synthetic mRGCs. Note that, for both subject optics, the synthetic mRGC $R_c$ follows the trend seen in macaque mRGCs, with good agreement at eccentricities above 10$^o$. In more central locations, however, the $R_c$ of the synthetic mRGCs is 2–3 times smaller than the $R_c$ measured in macaque mRGCs. We believe that the discrepancy at central locations is not a deficiency of our model, but rather results from several factors.

First, the cone mosaic in our model has a peak density of 288,000 cones/mm$^2$ which is near the high end of densities reported in humans (Curcio et al., 1990), whereas the average macaque peak cone density is around 200,000 cones/mm$^2$ (Packer et al., 1989; Schein, 1988). The higher cone density in humans implies smaller cone apertures, which in turn would bias our synthetic mRGCs towards somewhat smaller RF centers.

Second, in acute macaque experiments, the achieved optical refraction is not necessarily perfect, so there could be residual blur due to errors in refraction, as well as due to corneal edema from the contract lens used in typical multi-day acute experiments. This would increase the size of the RF centers in the physiological data relative to those in our model in central retina. Moreover, residual eye movements can occur in acute experiments, despite the ocular muscle paralysis (personal observations by N.P. Cottaris). Such residual movements would artificially enlarge estimates of RF center size for central retina mRGCs. Finally, in the macaque mRGC $\textrm{vSTF}$ characterizations of Croner & Kaplan, stimulus orientation was not optimized to match any orientation bias in the RF of macaque mRGCs (Lisa Croner, personal communication), whereas in the simulated experiments, stimulus orientation was matched to the cell’s visual-space referred orientation bias, which results in the smallest possible estimate of RF center size.

In additional analyses (not shown), we computed $\textrm{vSTF}$s using random grating orientations as well as a fixed orientation (as was done by Croner & Kaplan) for eccentricities between 1$^o$ and 8$^o$ along the nasal meridian. We found small effects of grating orientation on the estimates of $R_c$ in the direction of bringing the estimated $R_c$ into closer agreement with the values resported by Croner & Kaplan. None-the-less, the enlarged estimates still fall short of the reported values, so we think the first two factors mentioned above are likely to be important.

Further support for our assertion that the discrepancy in $R_c$ between synthetic and macaque mRGCs at central locations is not a deficiency of our model, is provided by *in vivo* data from foveal macaque mRGC $\textrm{vSTF}$s obtained under adaptive optics viewing conditions (Godat et al., 2022). The center sizes of these cells, blurred by the optics measured for the monkey subjects studied, are depicted by the purple disks in Fig. 8C1 & C2. Note that these align well with the $R_c$ values of our synthetic mRGCs.

**Fig. 9:**
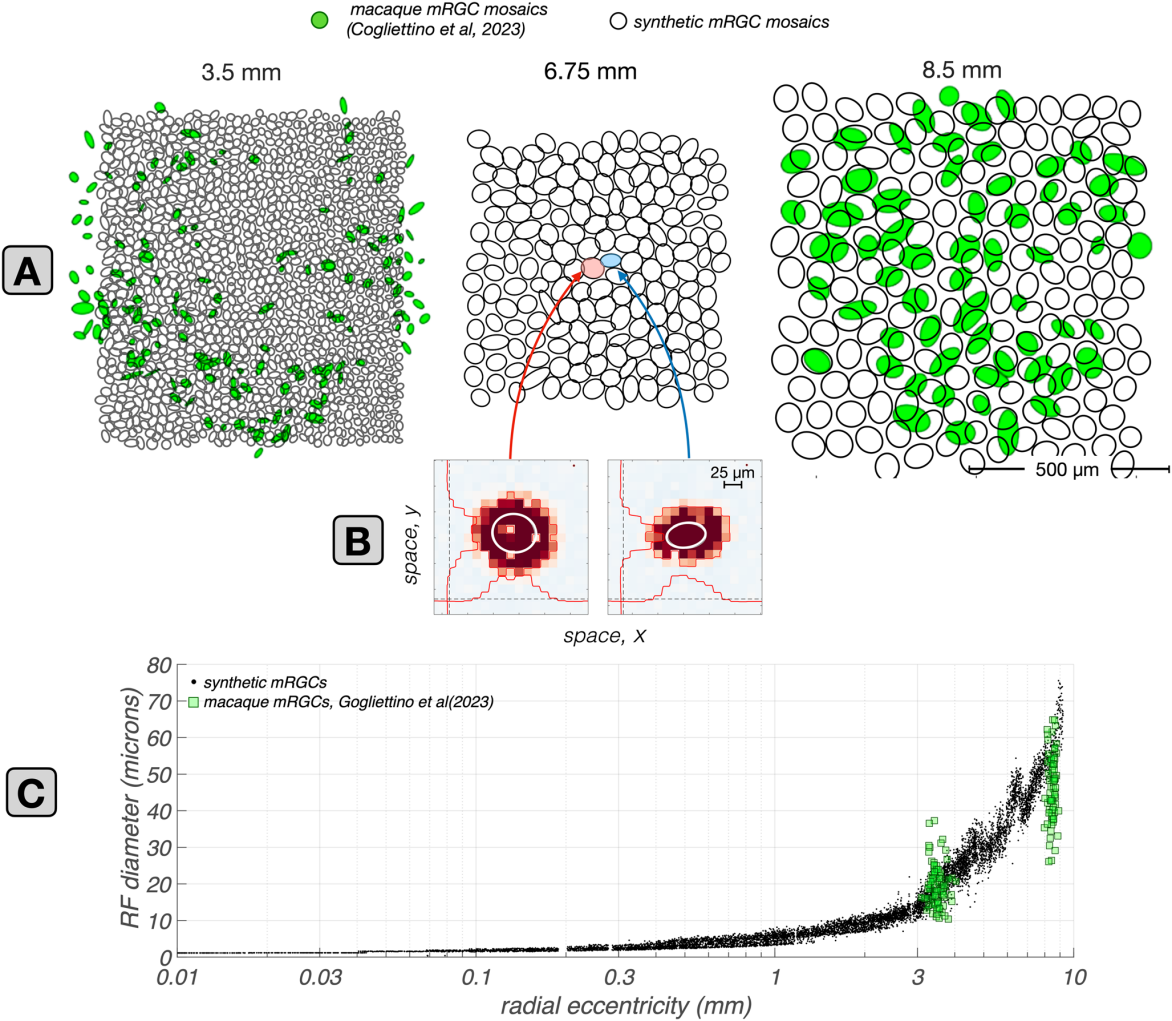
Retinal space–referred RF center sizes: synthetic vs. macaque mRGCs recorded *in vitro*. **A:** Mosaics of synthetic mRGCs synthesized at three eccentricities, 3.5, 6.75, and 8.5 mm along the temporal meridian. The black contours depict Gaussian ellipsoid fits to the increment-excitatory regions of the computed RF maps, drawn at the $e^{-1}$ normalized sensitivity level. Only the increment-excitatory region of the RF map is fitted. Green contours depict RF maps from two macaque mRGCs mosaics from the *in vitro* recordings of Gogliettino et al. (2023). **B:** Example spatial RF maps of two synthetic mRGCs located at 6.75 mm, computed via white noise stimulation delivered to the retina under diffraction limited optics. Regions excitatory to light increments, i.e. the RF centers, and to light decrements, i.e., the RF surrounds, are indicated by red and blue colors, respectively. The scattered zero excitation spots within the light-increment RF centers correspond to the location of S-cones. White lines depict iso-contour plots of Gaussian ellipsoids fitted to the light increment–excitatory RF center region, drawn at the $e^{-1}$ normalized sensitivity level. **C: ** Comparison of synthetic against macaque mRGC RF center sizes across eccentricity. Black dots depict the RF diameters of synthetic mRGCs, computed from the Gaussian ellipsoid fits as $2 \times \sqrt{\sigma _{minor} \times \sigma _{major}}$, and green squares depict the RF diameters of macaque mRGCs at the two eccentricities where the *in vitro* measurements are available

### Validation against *in vitro* physiology in the periphery

We also compared spatial RF properties of synthetic mRGCs against macaque data from *in vitro* mRGC recordings. Since the *in vitro* data are not subject to optical blur, they may be compared directly to the retinal-space characteristics of our model. Data of this sort are currently only available in the peripheral retina.

The first study considered is that of Gogliettino et al. (2023), in which the spatial RFs of mosaics of macaque mRGCs were mapped using white noise stimulation. To simulate their experiments, we probed synthetic mRGCs with white noise modulated achromatic checkerboard stimuli delivered to the retina under diffraction limited optics. To compute the spatial RFs of synthetic mRGCs, we cross-correlated the synthetic mRGC responses with the white noise stimulus sequence. Results of this analysis are depicted in Fig. 9.

In Fig. 9A the mosaics of spatial RFs in mRGC mosaics synthesized at three eccentricities, 3.5 mm, 6.75 mm and 8.5 mm, are depicted by the black ellipses. The superimposed green filled ellipses depict spatial RFs of macaque mRGCs at 3.5 mm and 8.5 mm from the study of Gogliettino et al.. Note that at both eccentricities, there is good correspondence in RF center size and coverage between the synthetic and the macaque mRGC mosaics. To quantify the retinal space–referred RF center sizes in synthetic mRGCs, we computed the diameter of their RF centers as $\displaystyle 2 \times \sqrt{\sigma _{\textrm{minor}} \times \sigma _{\textrm{major}}}$, where $\sigma _{\textrm{minor}}$ and $\sigma _{\textrm{major}}$ are the standard deviations of the fitted Gaussian ellipsoid along its minor and major axes.

The results of this analysis across eccentricity are depicted by the black dots in Fig. 9C, along with the RF center diameters of mosaics of macaque mRGCs located at 3.5 mm and 8.5 mm, which are depicted by the green squares. Note that the correspondence between synthetic and macaque data is excellent at 3.5 mm, whereas at 8.5mm, the RF diameters of the synthetic mRGCs are, on average, 30–40% larger than the RF diameters of macaque mRGCs. The deviation in RF center size at the far periphery may occur because human and macaque retinas differ somewhat in the periphery. For example, in the human retina, cone density does not change much for eccentricities $>5$mm, whereas in the macaque retina it continues to drop as eccentricity increases (Grunert & Martin, 2020). The RF size deviation we observe could be the result of a higher mRGC density in the peripheral macaque retina, relative to the human retina.

**Fig. 10:**
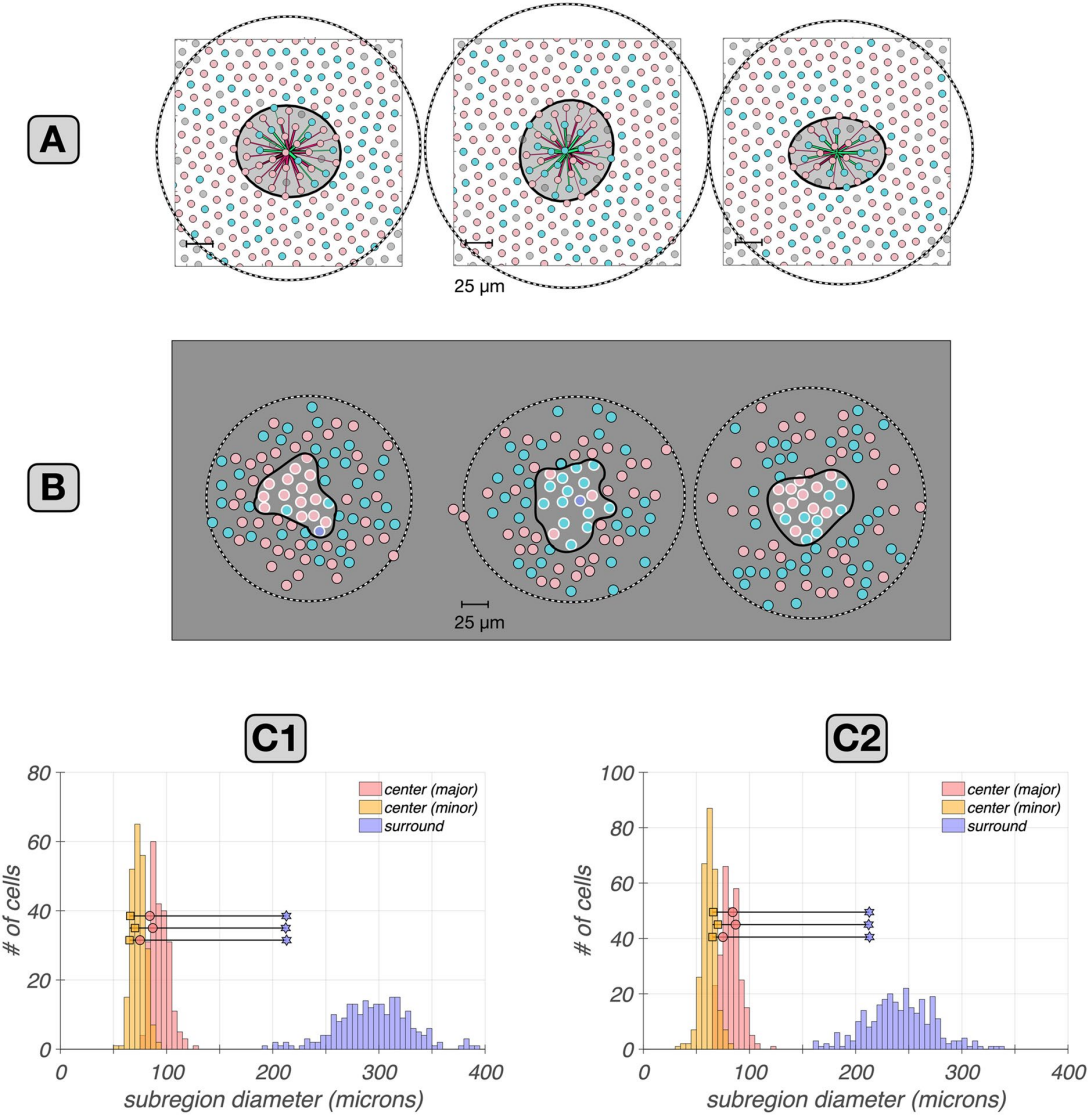
Cone pooling maps in RF centers and surrounds: synthetic vs. macaque mRGCs recorded *in vitro*. **A: ** Center and surround cone pooling weight maps for three synthetic mRGCs at an eccentricity of 6.75 mm along the temporal raphe. Solid and dashed contours include cones pooled by the RF center and the RF surround, respectively, with pooling weights $>0.005 \times$ the peak center weight. **B: ** Center and surround cone pooling weights for three macaque mRGCs recorded *in vitro* at an eccentricity of 6.75 mm along the temporal raphe. White and black disks indicate cones pooled by the RF center and the RF surround respectively, with same threshold pooling weights as in A. The macaque mRGCs are from the *in vitro* recordings of Field et al. (2010). **C1 &C2:** Comparison of minor and major diameters of the center pooling mechanism (yellow squares and pink circles) and of the surround pooling mechanism (purple stars) in the 3 macaque mRGC cells against corresponding distributions (yellow, pink and magenta histograms) in populations of synthetic mRGCs at eccentricities of 6.75 mm (C1) and 6.0 mm (C2)

The second *in vitro* study we validated our synthetic mRGCs against, is that of Field et al. (2010), which examined the spatial layout of single cone inputs to the RF centers and surrounds in peripheral macaque mRGCs. Results of this comparison are depicted in Fig. 10. Figure 10A depicts the cone pooling maps of three synthetic mRGCs at a temporal eccentricity of 6.75 mm. The spatial distribution of cone pooling weights in three macaque mRGCs at the same eccentricity from the study of Field et al. (2010), adapted from their Fig. 4, are shown in Fig. 10B. For both synthetic and macaque mRGCs, the visualized surround cones have pooling weights $>0.005~\times$ the peak center cone weight (Greg Field, personal communication).

Note the general agreement between synthetic and macaque mRGCs in the extent of both their RF centers and surrounds, although again, synthetic mRGCs appear to have slightly larger RFs that their macaque counterparts. Also notable is that the density of cones in the synthetic mRGC cone pooling maps is higher than that seen in the macaque mRGCs. This occurs because our model is based on human cone mosaics, and human cone density is higher than macaque cone density at temporal eccentricities above 5 mm (Grunert & Martin, 2020), which is where these comparisons are made.

To contrast the relationship in RF center and surround cone pooling regions between synthetic and macaque mRGCs more quantitatively, we compared the diameters of cone pooling regions of the three depicted macaque mRGCs against those of populations of synthetic mRGCs at two eccentricities: the 6.75 mm location at which the *in vitro* measurements were made, and a slightly less eccentric value of 6.0 mm. Results of this analysis are depicted in Fig. 10C1 and C2. The minor and major diameters of the RF center pooling mechanism and the diameter of the RF surround pooling mechanism for the 3 macaque mRGCs are depicted by the yellow squares, pink circles and magenta stars, respectively. The corresponding distributions in populations of synthetic mRGCs are depicted by the yellow, pink and magenta histograms. Note that at the 6.75 mm synthetic cell location (Fig. 10C1), the cone pooling regions of the synthetic mRGCs are larger than those of the measured macaque mRGCs. There is some uncertainty about how to best relate macaque and human retinal locations (see Methods), however, and at the slightly less peripheral eccentricity of 6.0 mm (Fig. 10C2) better agreement exists between model and macaque mRGCs. Measurements of human cone density at 6.0 mm of retina (Curcio et al., 1990) are also better matched to measurements of monkey cone density at 6.75 mm (Packer et al., 1989) than are human measurements at 6.75 mm.

These observations highlight an inherent issue in building our mRGC model, namely that we had to employ a mixture of human and macaque data sources: human data regarding the density of cones and the density of mRGC RFs across visual space, human data regarding the characteristics of physiological optics across the retina, and macaque data regarding the spatial characteristics of mRGC RFs and of H1 horizontal cells, with our validations done against macaque data. This is not ideal, as there are some differences between human and macaque retinas (Grunert & Martin, 2020). But, it is unavoidable given the lack of complete data in either species. The modeling framework that we devised however, which incorporates data from different sources, can be easily modified as new data become available.

**Fig. 11:**
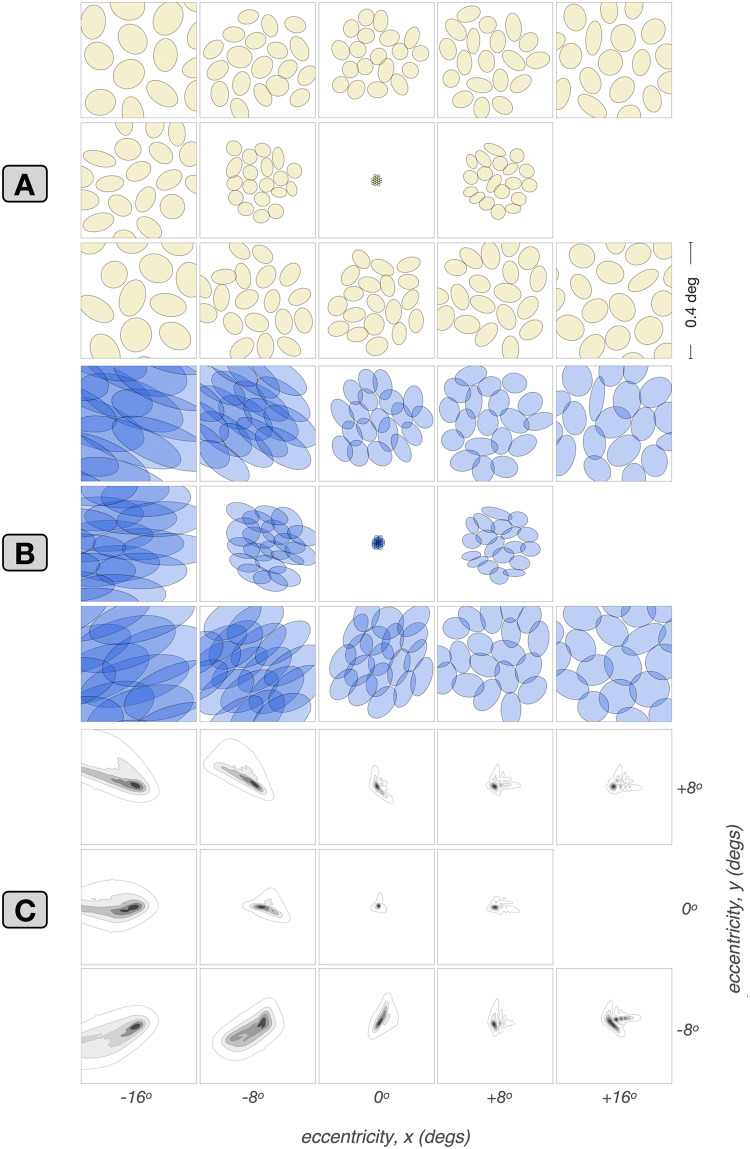
Retinal vs. visual space–referred mRGC RF maps across the retina. Illustration of the effect of physiological optics on visual space–referred spatial RF maps of synthetic mRGCs across eccentricity. **A: ** Retinal space–referred spatial RF maps at different (x,y) eccentricities. Within each panel, yellow contours depict Gaussian ellipsoid fits to RF maps of up to 19 cells from a single mRGC mosaic. RF maps are computed using white noise stimulation under diffraction limited optics. **B:** Visual space–referred spatial RF maps of the same cells, computed under physiological optics of one human subject at corresponding eccentricities. **C:** Point spread functions of the employed physiological optics at corresponding eccentricities

### Visual *vs.* retinal space– referred RFs: the impact of physiological optics

In this section we characterize how physiological optics interacts with the retinal cone pooling within the RFs of mRGCs to shape their visual space–referred RF properties. Figure 11 illustrates examples of this interaction at five horizontal eccentricities, $x = [-16^o, -8^o, 0^o, +8^o, +16^o]$, and 3 vertical eccentricities, $y = [-8^o, 0^o, +8^o]$. The yellow ellipses in each panel of the $3\times 5$ grid of Fig. 11A depict Gaussian ellipsoids fitted to the retinal space–referred RF maps of synthetic mRGCs at the examined eccentricities. The small and non-systematic orientation biases in the retinal space–referred RF maps emerge due to the pooling of multiple cones by the RF center mechanism and are reminiscent of RGC mosaics mapped *in vitro* (Gauthier et al., 2009).

The blue ellipses in Fig. 11B depict Gaussian ellipses fitted to the visual space–referred RF maps of the same cells. Note that there are striking and systematic orientation biases in these visual space–referred RF maps, which emerge due to the characteristics of physiological optics, whose PSFs are depicted in Fig. 11C. Clearly, the shape of the PSFs, especially at peripheral locations is a major determinant of the visual space–referred RFs in mRGCs.

Overall, this analysis demonstrates that there can be substantial differences between *in vivo* and *in vitro* estimates of the spatial RFs of mRGCs, and, once again highlights the notion that inferences regarding retinal wiring from *in vivo* measurements must be evaluated in the context of the effect of the physiological optics. Indeed, in recent on-going work (Cottaris et al., 2024), we have shown the importance of such analyses in assessing inferences regarding cone wiring to the surround subregions of mRGCs based on *in vivo* measurements of their spatio-chromatic RFs.

### Validity of the difference of Gaussians model applied to *in vitro* responses of mRGCs in retrieving their spatial pooling characteristics

In our synthetic mRGCs, the spatial characteristics of cone pooling within the RF center and the RF surround *component* mechanisms are known by design. This allows us to test how well one can predict these characteristics from DoG model fits to *in vitro* measurements of mRGC $\textrm{STF}$s, where the RF center and surround mechanisms are driven simultaneously, and in the absence of optics (Wool et al., 2018).

**Fig. 12:**
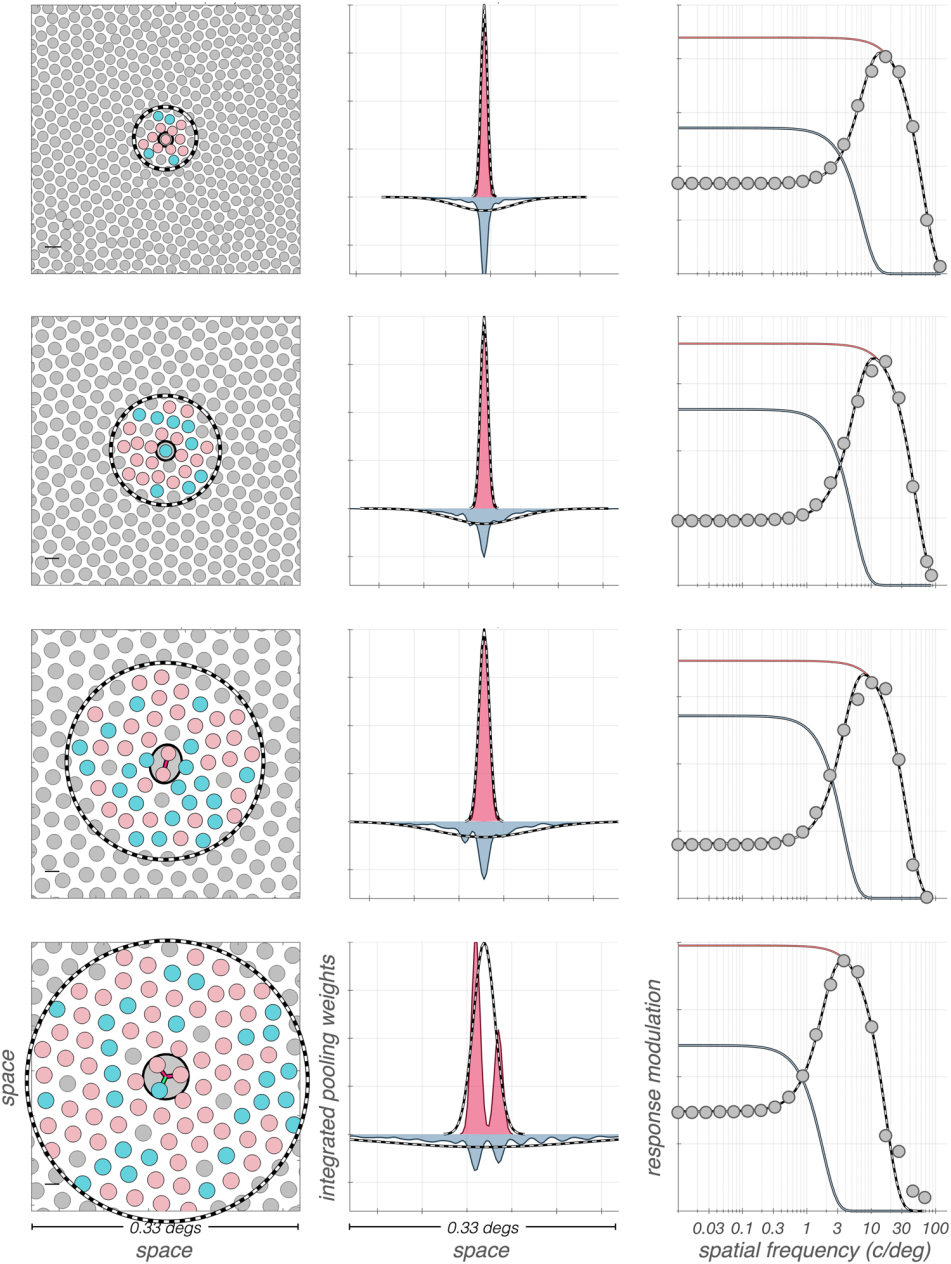
Spatial characteristics of mRGC surround cone pooling inferred from DoG model fits to retinal space–referred $\textrm{vSTF}$s are not accurate. The correspondence between actual and inferred surround cone pooling is examined for exemplar synthetic mRGCs at four eccentricities. **Left panels:** Cone input maps of RF center and RF surround mechanisms. **Middle panels:** Line weighting functions of cone inputs pooled by the RF center and the RF surround mechanisms, depicted by pink and slate histograms, respectively. Dashed lines represent line weighting functions as inferred from DoG model fits to the cells’ retinal space–referred STFs. **Right panels:** Retinal space–referred STFs, depicted by gray disks, computed for stimuli delivered to the retina via diffraction limited optics. The DoG model fits to the computed STFs are depicted by the dashed black lines, with the corresponding center and surround components depicted by the pink and slate lines, respectively

Results of this analysis are illustrated in Fig. 12. The cone pooling maps of four exemplar mRGCs are depicted in the left column. The cells in the top two rows both have RF centers with a single cone input, whereas the cell in the third row has a 2-cone RF center, and the cell in the fourth row has a 3-cone RF center. The pink and maroon histograms depicted in the middle column of Fig. 12, are the y-axis integrated cone pooling weights within these cells’ RF center and surround subregions, respectively. The superimposed dashed lines depict the center and surround line weighting profiles, as estimated by the DoG model fit to the cells’ retinal space–referred STFs, which are depicted by the gray disks in the right column of Fig. 12.

Note that although the DoG model fits to the computed retinal space–referred STFs (dashed lines in right column) are good for all cells, the inferred spatial RF profiles, (dashed lines in the middle column) do not capture accurately the cone pooling regions of the RF surrounds (slate histograms in the middle column). The discrepancy between actual and inferred surround pooling is most obvious in the two top cells which have single-cone RF centers, and becomes less pronounced as RF center size increases. The discrepancy involves both the spatial extent and the peak sensitivity of the inferred surround pooling, which is estimated by the DoG model to be more diffuse with a weaker peak sensitivity than the cell’s actual surround cone pooling.

It is perhaps not surprising that the DoG model does not do a good job of fitting the model cell surrounds, given that they were constructed as double exponentials to match the spatial properties of H1 horizontal cells. The key point, however, is that the DoG model fits to the observable composite STFs are quite good. These observations suggest that caution should be exercised when inferring mRGC RF surround properties from DoG model fits to *in vitro* STF measurements.

### Applications

We (Cottaris et al., 2019; Cottaris et al., 2020; Zhang et al., 2022) and others (Banks et al., 1987; Geisler, 1989; Sekiguchi et al., 1993) have reported on how the representation of visual information at the level of the cone mosaic shapes visual performance, in our case by exploiting the ISETBio image computable model of cone excitations. The transformation from cone excitations to RGC responses further shapes the information available for perceptual decisions, and we can interrogate our linear spatio-chromatic RF model of the ON–center mRGC mosaic to understand how the information available from this neuron class differs from that at the cone mosaic.

In this section, we present two example computations of this nature. Our goal is to illustrate how our model may be exploited in this way, and not to present a full analysis in either case. Even these initial calculations, however, provide interesting insight.

#### Achromatic and chromatic spatial contrast sensitivity

We used a computational observer approach to compute spatial contrast sensitivity functions (CSFs) for achromatic and $\mathrm {L-M}$ cone opponent stimuli, based both on the representation at the cone mosaic and on the representation at the mRGC mosaic. To do so, we computed responses to drifting gratings of varying spatial frequency, $\omega$.

For the achromatic gratings, the L–, M–, and S–cone contrast component gratings were in phase, $C_L(\omega , x, y) = C_M(\omega , x, y) = C_S(\omega , x, y)$. For the $\mathrm {L-M}$ gratings, the L– and M–cone contrast components were in antiphase, $C_L(\omega , x, y) = -C_M(\omega , x, y)$, and $C_S(\omega , x, y) = 0$. For all stimuli, the mean (*x*, *y*) chromaticity was (0.30, 0.32) and the mean luminance was 100 $cd/m^2$. Stimuli were simulated as presented on a typical CRT monitor, but with 20-bit channel DACs, to avoid intrusion of quantization effects.

For each eccentricity we studied, we oriented the gratings so that they were aligned with the axis of elongation of the optical point spread function at that eccentricity. Stimulus size was specified so that it extended over the area spanned by the input cone mosaic of the employed mRGC mosaic. The size of the mRGC mosaics was varied between eccentricities so as to achieve nearly equal numbers of mRGCs for mosaics between which we wished to compare performance.

Cone fundamentals vary with eccentricity because of variation in macular pigment density and photopigment axial density, and this variation is captured by ISETBio. Therefore, in these computations, stimuli were designed using cone fundamentals specific to the eccentricity of the employed mRGC mosaic.

At present, our mRGC model does not include spike generation or response noise. Therefore, in the computations described here we modeled response variability by adding zero mean Gaussian noise to the noise-free responses of the synthetic mRGCs. This approximation allows us to examine relative sensitivity across stimuli and eccentricity, but the overall level of predicted sensitivity is arbitrary. Given the choice of Gaussian noise, we used a template matching computational observer decision rule, with templates provided by the noise-free mRGC responses to the stimuli being discriminated. For comparing computational observer performance at the mRGCs with that at the cones, we also adopted the Gaussian noise approximation for the cone excitations, and used the template matching decision rule.

To estimate contrast sensitivity, we varied, for each tested spatial frequency, $\omega$, the contrast of the test stimulus and identified threshold contrast, $\mathrm C_{\textrm{threshold}}(\omega )$, as that for which the probability of correctly identifying the test versus a zero contrast stimulus was 80.6%. Contrast sensitivity was defined as $\textrm{CSF}(\omega ) = 1/\textrm{C}_{\textrm{threshold}}(\omega )$.

**Fig. 13:**
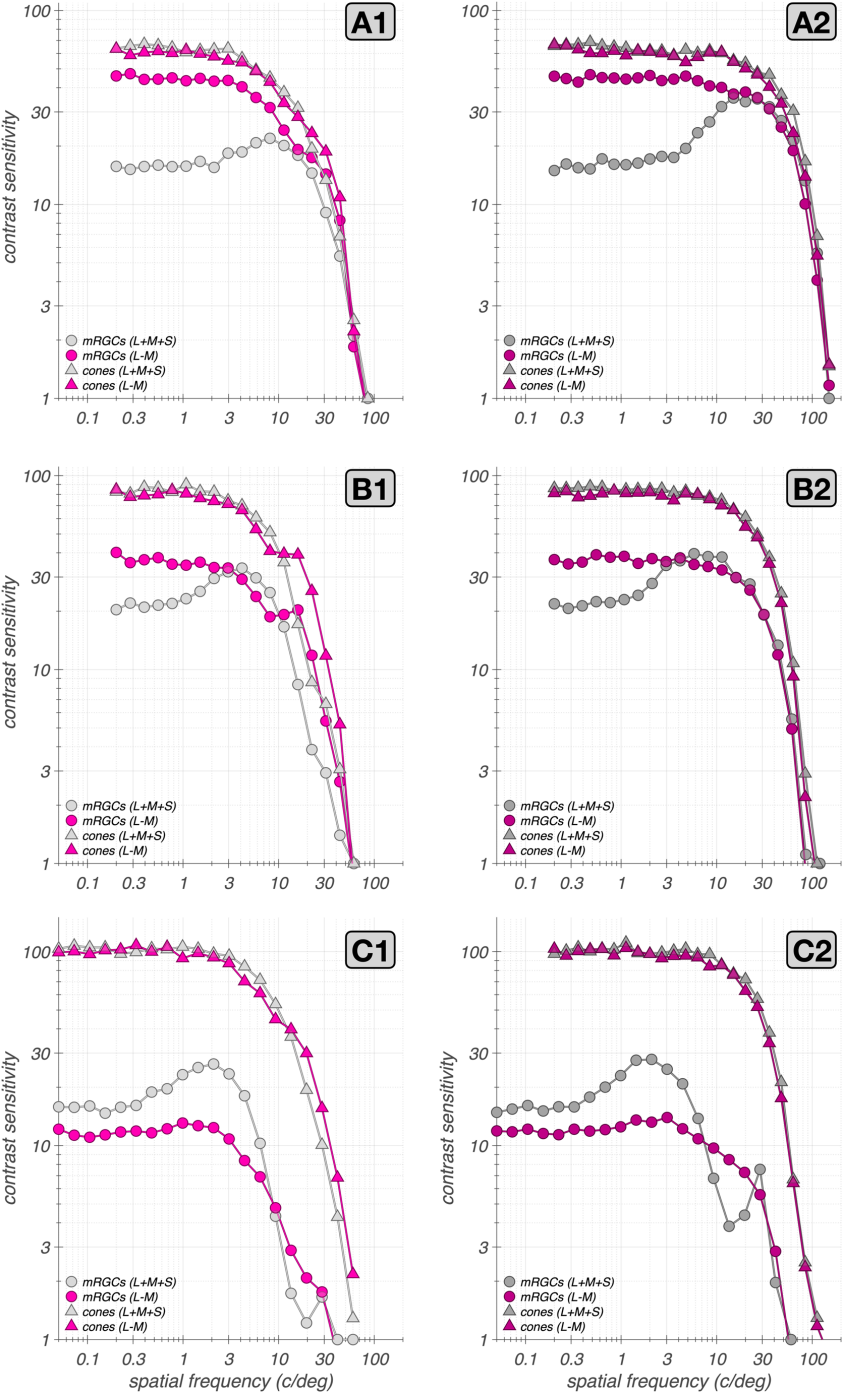
Computational observer spatial CSFs. Left column: CSFs computed with stimuli that are delivered to the retina through typical human optics. Right column: CSFs computed with stimuli that are delivered to the retina under diffraction limited optics. Disk and triangles depict the CSFs of the mRGC mosaic and of its input cone mosaic, respectively. Gray and magenta symbols depict achromatic and $\mathrm {L-M}$ CSFs, respectively. **A1&A2:** CSFs for a $0.6^{\circ } \times 0.6^{\circ }$ foveal mRGC mosaic and of its input cone mosaic. This mosaic contains 4628 mRGCs. **B1&B2:** CSFs for a $2.1^{\circ } \times 2.1^{\circ }$ parafoveal mRGC mosaic synthesized at an eccentricity of 4$^\circ$ along the temporal meridian. This mosaic contains 4633 mRGCs. **C1&C2:** CSFs for a $4.1^{\circ } \times 4.1^{\circ }$ peripheral mRGC mosaic synthesized at an eccentricity of 14$^\circ$ along the temporal meridian. This mosaic contains 2195 mRGCs

Estimates of so computed contrast sensitivities at three eccentricities are depicted in Fig. 13. The contrast sensitivities for stimuli viewed through typical human optics are shown in the left panels of Fig. 13, with disks and triangles depicting sensitivity at the mRGC mosaic and at its input cone mosaic, respectively. For comparison, the right panels of Fig. 13 depict corresponding calculations for stimuli viewed under diffraction-limited optics with no chromatic aberration, as might be measured using adaptive optics. The comparison between left and right panels helps understand which effects in the computed CSFs have their origin in the optics or sampling by the cone mosaic, and which should be attributed to retinal processing through to the mRGCs.

At the fovea, the CSFs at the cone excitation level (triangles in Fig. 13A1), are low pass for both achromatic and $\mathrm {L-M}$ stimuli. This is expected because there is no spatial antagonism at the level of the photopigment excitations, and because we do not incorporate spatio-temporal coupling that arises because of interactions between fixational eye movements and post-receptoral temporal filtering (Casile et al., 2019; Houston et al., 2025).

On the other hand, the achromatic CSF at the mRGC mosaic exhibits a mild low-spatial frequency attenuation, which is due to the spatial antagonism between the RF centers and surrounds. Note that the low frequency attenuation appears weaker than what is observed under diffraction limited optics (Fig. 13A2). This occurs because physiological optical blur carves sensitivity at the high frequency regime, thereby reducing the apparent effect of the mRGC surrounds on the CSF. We observed a similar effect in foveal macaque mRGCs whose responses were measured under adaptive optics conditions (Godat et al., 2022).

The $\mathrm {L-M}$ opponent CSF of the mRGC mosaic lacks the low-frequency attenuation seen for achromatic modulations because in foveal mRGCs, $\mathrm {L-M}$ cone opponent stimuli do not induce substantial spatial antagonism between their single cone RF centers and their surrounds. These observations, which are consistent with what is known regarding the $\mathrm {L-M}$ chromatic contrast sensitivity of the mRGC pathway (Mullen, 1985; Wool et al., 2018), demonstrate that $\mathrm {L-M}$ sensitivity exceeds achromatic sensitivity at low spatial frequencies, consistent with the literature (Chaparro et al., 1993).

At high spatial frequencies there is little difference between computational observer sensitivity to achromatic and $\mathrm {L-M}$ modulations. This is not true of human observers, where sensitivity drops more rapidly as a function of spatial frequency for red-green isoluminant gratings than for achromatic gratings, either with (Mullen, 1985) or without typical optical blur (Sekiguchi et al., 1993). Although our $\mathrm {L-M}$ opponent CSFs are not precisely equivalent to the red-green isoluminant CSFs measured in many human experiments, this is not the primary source of the difference between computational and human observers. Rather, it is known that compared to ideal observers, humans lose foveal information available from the cones more rapidly as a function of spatial frequency for red-green than than for achromatic gratings (Sekiguchi et al., 1993). Our example calculation here suggests that this information loss should not be attributed to the linear receptive fields of the mRGCs.

We believe this is because optical blur dominates computational observer performance at high spatial frequencies and the single cone RF centers of foveal mRGCs transmit information about each type of stimulus equally well; the surrounds have little effect at high spatial frequencies. Also, we do note that, in the present calculations, the specific resolution limit, i.e., the spatial frequency at which sensitivity drops to 1, depends on the variance of the added Gaussian noise and is thus somewhat arbitrary. We have chosen a noise level that is low relative to human observers so that our computations show the behavior in the high-spatial frequency regime more fully than would psychophysics conducted through natural optics.

As we move to more peripheral locations, additional features of the CSF emerge. Figure 13B1 and C1 depict results of computations at 4$^\circ$ along the temporal meridian. Note that under physiological optics viewing (Fig. 13B1) there is a spatial frequency regime in which $\mathrm {L-M}$ sensitivity exceeds the corresponding achromatic sensitivity, with the $\mathrm {L-M}$ CSF having a notched shape. We have reported this observation in conference abstract form (Hong et al., 2024). It occurs because of the wavelength dependent defocus that is introduced by longitudinal chromatic aberration (LCA), which can change the spatial phase of the L– and M–cone stimulus components in the retinal image. Consistent with this interpretation, the notch is present in the CSFs both at the cones and at the mRGCs on the left, but not under diffraction-limited optics (Fig. 13B2), where LCA is zero. Similar effects have been observed for S–cone CSFs (Cruz et al., 2019). We have presented in abstract form experimental results that suggest that these effects occur in measurements of the human $\mathrm {L-M}$ spatial CSF (Oh et al., 2025).

Comparison of the cone–based CSFs in Fig. 13A1 with those in Fig. 13B1 and C1 reveals the effect of stronger optical blur with eccentricity, which increases the rolloff of the CSFs with spatial frequency. Similar comparison of the mRGC–based CSFs shows additional rolloff introduced by the increasing size of mRGC RF centers with eccentricity.

Additional observations are notable at 14$^{\circ }$ (Fig. 13C1 and C2). First, a notch arises in the achromatic CSF at high spatial frequencies for the mRGC CSF that is not apparent in the cone CSF. This seems unlikely to be an optical effect, because it is more salient in Fig. 13C2 where optical effects are not present. To explore the origin of this effect, we computed CSFs at different orientations (not shown), which show that this notch is orientation dependent and has to do with the precise alignment of individual cones within the receptive field centers of mRGCs. We do not explore it further here.

We note that our computational observer is with respect to an RGC noise level that may make it more sensitive than the human observer. If so, the notches shown in Fig. 13 might not be revealed with psychophysics. In further simulations (data not shown) conducted with twice the noise variance, we observed that, in addition to an overall reduction in sensitivity, the high frequency notches disappeared below the sensitivity floor. Therefore, the effects shown in Fig. 13, if they exist, are most likely to be revealed under conditions that maximize psychophysical sensitivity (i.e., bright adapting background, stimuli that fill the spatial and temporal integration area and duration, adaptive optics viewing).

Finally, note that the $\mathrm {L-M}$ advantage over the achromatic CSF is reversed at 14$^{\circ }$ of eccentricity. This is because at such high eccentricities, the $\mathrm {L-M}$ signal is reduced by the increased mixing of L– and M–cone signals within the larger mRGC RF centers and surrounds. Careful comparison of this effect with computational observer predictions for various choices of the model’s spatial homogeneity/spectral purity tradeoff parameter, $\phi$, is an interesting future direction.

#### Chromatic contrast sensitivity of synthetic mRGC mosaics: dependence on eccentricity

As a second example application, we examined chromatic sensitivity for uniform fields modulated along different directions in the L/M–cone contrast plane. We used the same computational observer approach described above, and evaluated threshold for stimuli whose contrast was modulated in time. The cone contrasts of stimuli at different chromatic directions, $\theta$, on the LM plane were: $C_L(\theta ) = \rho \cdot \cos (\theta ); ~C_M(\theta ) = \rho \cdot \sin (\theta ); ~C_S(\theta ) = 0$. For each $\theta$, we varied $\rho$ to find its threshold value for discriminating that modulation direction from a zero contrast stimulus with a probability of 80.6%. To summarize the computed thresholds across the different chromatic directions, we fit ellipses to the locus of threshold contrast points.

**Fig. 14:**
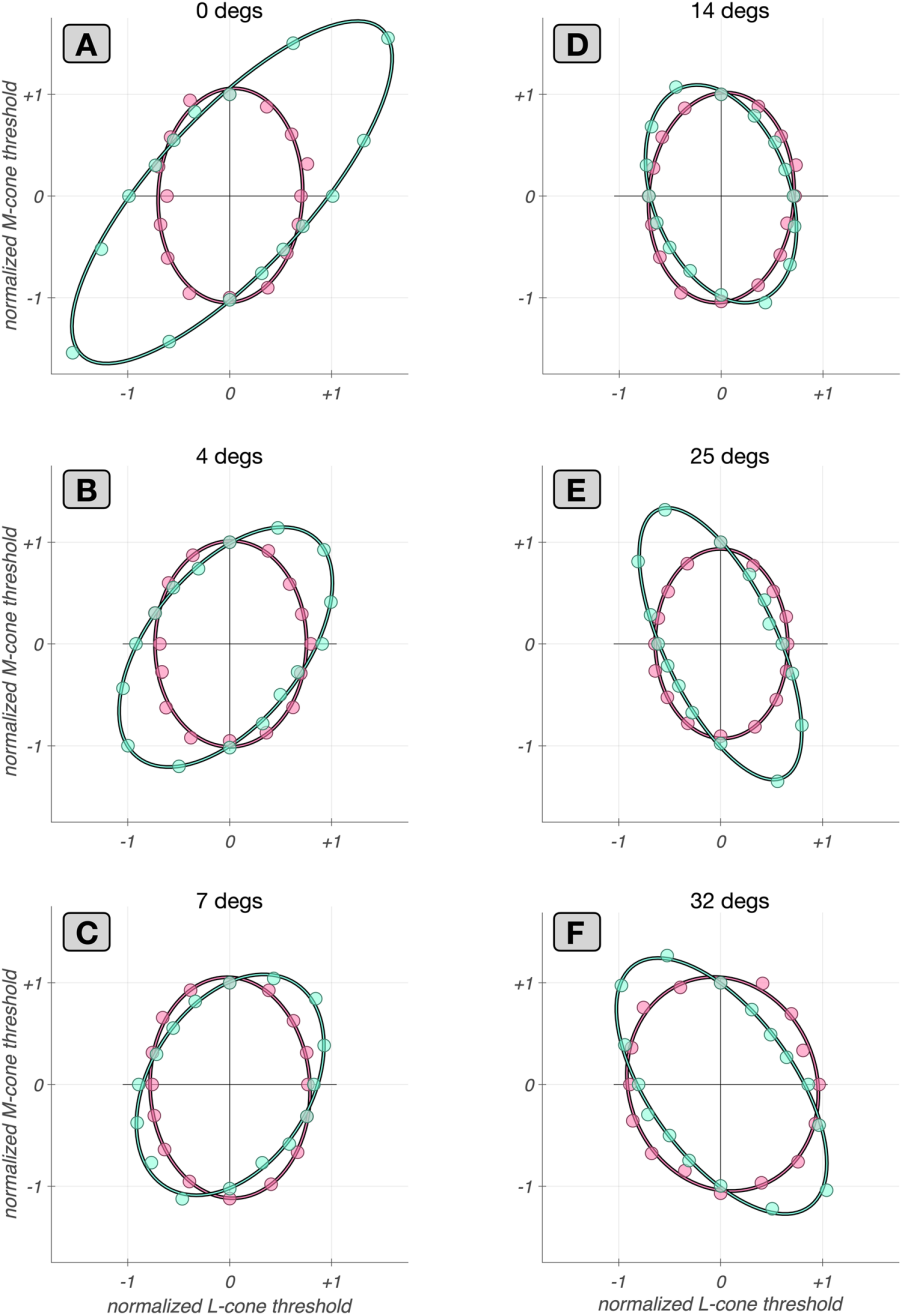
Chromatic contrast sensitivity of synthetic mRGC mosaics: dependence on eccentricity. Discrimination thresholds along the L/M–cone contrast plane of mRGC mosaics (green disks) and of their input cone mosaics (pink disks), computed for uniform field stimuli (0 c/deg). **A:** Data from a $0.6^{\circ } \times 0.6^{\circ }$ foveal mRGC mosaic. **B:** Data from a $2.1^{\circ } \times 2.1^{\circ }$ parafoveal mRGC mosaic synthesized at an eccentricity of 4$^\circ$ along the temporal meridian. **C:** Data from a $3.2^{\circ } \times 3.2^{\circ }$ parafoveal mRGC mosaic synthesized at an eccentricity of 7$^\circ$ along the temporal meridian. **D:** Data from a $4.1^{\circ } \times 4.1^{\circ }$ peripheral mRGC mosaic synthesized at an eccentricity of 14$^\circ$ along the temporal meridian. **E:** Data from a $6^{\circ } \times 6^{\circ }$ peripheral mRGC mosaic synthesized at an eccentricity of 25$^\circ$ along the temporal meridian. **F:** Data from a $9^{\circ } \times 9^{\circ }$ peripheral mRGC mosaic synthesized at an eccentricity of 32$^\circ$ along the temporal meridian

Figure 14 depicts computational observer thresholds for synthetic mRGC mosaics and for their input cone mosaics at different eccentricities. Note that how computational observer sensitivity changes with eccentricity depends on how stimulus size is covaried with eccentricity, as does human sensitivity (e.g. Hansen et al., 2009). Comparison of the magnitude of sensitivity for cone– and mRGC–based computational observers depends on how the noise levels are chosen. For these example calculations, we focus on the shape rather than magnitude of the elliptical threshold contours. Therefore, each contour shown in Fig. 14 is normalized so that the threshold along the M cone direction is equal to one.

A few observations are notable. First, the normalized contours for the cone mosaic–based observer are similar across eccentricities and align with the L– and M–cone contrast axes. They are more elongated in the M–cone direction because our mosaics have more L cones than M cones. The alignment with the axes is expected (Stockman & Brainard, 2010), and the similarity of the normalized shapes occurs because this shape depends primarily on the relative numbers of L and M cones.

Second, in contrast to the cone mosaic–based thresholds, the mRGC mosaic–based threshold contours change markedly with eccentricity. For the foveal mRGC mosaic, the threshold ellipse is highly elongated along $45^\circ$ in the L/M–cone contrast plane, indicating that the highest discrimination thresholds occur when $C_L = C_M$ and lowest thresholds occur when $C_L = -C_M$. This difference in comparison to the cone–based computations is a consequence of the chromatic–opponency of foveal mRGC RFs, which have single cone centers, and thus opponency between their centers and the surrounds as the surrounds draw on mixed cone-types (Lennie et al., 1991; Mullen & Kingdom, 1996). This opponency leads to cancellation of non–opponent L– and M–cone signals for low spatial frequency stimuli and thus the observed contour elongation along $45^\circ$ (Stockman & Brainard, 2010; Wandell, 1995).

Third, as eccentricity increases, the contours first become less elongated and then elongation starts increasing again but along the $135^\circ$ rather than the $45^\circ$ axis. This is because the cone non–selective wiring model we implemented leads to progressively less opponency with increasing RF center size (Lennie et al., 1991; Mullen & Kingdom, 1996; Wool et al., 2018).

Although the qualitative features that emerge from this example calculation are understood in the literature, the example illustrates that our model enables this type of calculation to be made quantitatively in a way that takes chromatic aberration, stimulus size, spatial frequency, and retinal position into account. Of particular interest to us will be exploring how this type of threshold contour varies with the tradeoff between spatial homogeneity and spectral purity of mRGC RF centers (the center wiring parameter $\phi$ of our model).

## Discussion

We developed an image computable model of the linear spatio-chromatic RF mosaic of mRGCs across the retina. The model extends our image-computable cone mosaic model (Cottaris et al., 2019, 2020) by adding a layer of mRGCs which pool signals directly from the cone mosaic. The connectivity between cones and mRGCs is derived using a simulation framework that integrates anatomical, physiological and optical quality data, all of which vary across eccentricity.

By explicitly modeling the optics and photoreceptors, rather than directly expressing the RFs in terms of the stimulus, we are able to link our model with both *in-vitro* and *in-vivo* data, and to make predictions over a range of experimental conditions that are otherwise difficult to compare. These include psychophysical and physiological measurements made through physiological optics (natural viewing conditions), interferometric and adaptive optics techniques that bypass or correct for optical aberrations, and *in-vitro* physiology, where the natural optics are not present.

To build the model we had to overcome the challenge that current data about mRGC properties are incomplete and, where they exist, may come from different species, different measurement modalities, and from different eccentricities. For example, there are *in-vivo* measurements of mRGC linear receptive fields across the retina (Croner & Kaplan, 1995), but physiological optics blur the stimuli so that they do not constrain mRGC input at the cone-by-cone resolution we seek. On the other hand, although there is single cone-resolution connectivity data from *in-vitro* physiology (Field et al., 2010), these data are currently limited to large eccentricities ($\ge 25^o$). Thus, we developed a modeling framework that allows integration of data from multiple sources. This framework is an important contribution in its own right; we expect it will be useful to us and others, for incorporating new data that become available and for modeling other RGC classes.

We showed that the model captures visual space–referred spatial RF properties of macaque mRGCs recorded *in-vivo* across eccentricities, as well as retinal space–referred spatial RF properties of macaque mRGCs recorded *in-vitro*. We also showed that physiological optics plays a major role in shaping the visual space–referred spatial RF properties, so that inferences regarding retinal circuitry made from *in-vivo* measurements need to be evaluated in the context of the optics. Further, we showed that even under *in-vitro* conditions, where the optics are eliminated, the traditional approach of fitting a Difference of Gaussian model to spatial responses can lead to incorrect assessments of the properties of cone pooling in the mRGC surrounds.

### Applications

We employed an early version of the current model to interpret measurements of foveal macaque mRGCs measured *in-vivo* using adaptive optics (Godat et al., 2022). Specifically, the model allowed us to relate the adaptive optics measurements to *in-vivo* measurements conducted under physiological optics. For this purpose, the ability to move back and forth between retinal and visual space–referred representations was critical.

We are currently employing the model to assess inferences regarding the wiring of cone inputs to mRGC RF surrounds based on spatial RF measurements conducted *in-vivo* (Reid & Shapley, 1992). Specifically, we are analyzing the substantial effect that chromatic aberration plays in shaping mRGC responses to cone isolating stimuli, and how these effects can help reconcile tension between results from *in-vivo* physiology on the one hand and results from anatomy and *in-vitro* physiology on the other (Cottaris et al., 2024).

In parallel on-going work, we deploy the model to understand how the spatio-chromatic properties of the ON–center mRGC mosaic influence the information available for human spatio-chromatic vision, by applying computational observer analyses to the mRGC representation we compute (Hong et al., 2024; Oh et al., 2025). Although additional model components will influence this representation, for threshold tasks, where the stimulus perturbations are small, we expect the linear approximation to hold sufficiently well that the results will be informative.

In this work, we presented examples of this type of computation, to illustrate how the representation at the mRGCs differs from that at the cone mosaic and how this varies with eccentricity.

### Limitations and future directions

We conclude with discussing the various limitations of the model in its present state and our plans for augmenting the model to increase its realism.

#### Human versus macaque

When available, we used human data to guide model development, in order to maximize the usefulness of the model in predicting human performance. Even if this had not been our goal, we would have had to bring in human data to characterize the physiological optics across the visual field, as such data are not currently available in macaque. At the same time, not all the required data are available for human: although measurements of cone and mRGC density and physiological optics across the retina are available, physiological characterizations come from the macaque.

The need to mix data across the two closely related species produces tension in cases where the parameters for the two species differ. An example is the different cone densities in the far periphery (Grunert & Martin, 2020), which intrudes on the interpretation of the comparison between our model and *in-vitro* physiology in that retinal region. As more data become available in both species, and as species differences come more fully into focus (Kim et al., 2023), our approach should allow more fully differentiated models to be developed targeted at each.

#### Noise, nonlinearities and temporal dynamics

Although the current model captures fundamental aspects of the visual representation at the level of the mosaic of ON–center mRGCs, there are known characteristics of mRGCs that it does not account for. These include static and spatial nonlinearities, temporal filtering, spike generation, and physiologically constrained response noise. The modeling framework we developed is extensible however, so that these components may be included through future work.

Response variability models are available for macaque mRGCs, as descriptions of spike generation mechanisms (Croner et al., 1993; Pillow et al., 2005; Sun et al., 2004). In addition, we can incorporate nonlinearities, such as (a) light adaptation effects introduced through the phototransduction cascade (Angueyra & Rieke, 2013), (b) compressive and expansive static nonlinearities in the output of mRGCs (Pillow et al., 2005; Raghavan et al., 2023), and (c) spatial nonlinearities introduced by rectifying sub-units within the RFs of mRGCs (Freeman et al., 2015; Hong & Rieke, 2024). Explicit inclusion of photocurrent-based responses in the input to the mRGCs introduces a temporal component to the response model (Angueyra & Rieke, 2013). In addition, a second temporal filter may be added, such that when combined with the photocurrent filter will yield the bandpass filter characteristics observed in macaque mRGCs (Benardete & Kaplan, 1997).

Our current model does not represent explicitly the properties of the retinal circuitry (horizontal, bipolar, and amacrine cells) that produces the mRGC response properties, as we have opted instead to work towards a functional model that describes those properties. A complementary mRGC modeling approach that does consider some of these cell types explicitly has recently been published (Somaratna & Freeman, 2025), and there are other modeling efforts that have examined the influence of the various retinal interneurons on RGC response properties (Hennig et al., 2002; Wohrer & Kornprobst, 2009). We note however, that some of the processing performed by these other retinal cell types is incorporated implicitly in the current cone-to-mRGC model, such as the parametric form of the surrounds inherited from H1 cells.

The framework we developed is designed so that it would be possible to interpose explicit models of intermediate retinal cell types. Representing the action of different cell types explicitly may in the longer run be an effective way to account for response nonlinearities in the mRGCs, or in other classes of retinal ganglion cells. Moreover, using our framework to model other cell classes may be of interest to those seeking to interpret responses of those classes *per se*, or in the retinal mechanisms that produce RGC response properties.

**Table 1:** List of tutorials for computing with existing mRGC mosaics and for *de novo* synthesis of mRGC mosaics

Tutorial name	Scope
*Computing with mRGC mosaics*	
t_mRGCMosaicVisualizeWithOptics.m	Visualizes a previously synthesized mRGC mosaic and the optics that were used for its synthesis
t_mRGCMosaicInspect.m	Visualizes an mRGCMosaic and cone pooling maps of individual cells
t_mRGCMosaicBasicComputation.m	Performs a basic computation with an mRGC mosaic
*Synthesizing mRGC mosaics*	
t_mRGCMosaicSynthesizeAtStage1.m	Denovo synthesis of the spatial position lattices of cones and mRGC RF centers (stage 1)
t_mRGCMosaicSynthesizeAtStage2.m	Denovo synthesis of an mRGC mosaic at different sub-stages of cone-to-mRGC RF center connectivity (stage 2)
t_mRGCMosaicSynthesizeAtStage3.m	Denovo synthesis of an mRGC mosaic at different sub-stages of cone-to-mRGC RF surround connectivity (stage 3)

#### OFF mRGC mosaic

Because we model the linear RF, the distinction between ON and OFF mRGCs is subtle. However, our model should be thought of as a model of only the ON–center mRGCs because the synthetic cells only pool signals from L– and M–cones. This is believed true for ON–center mRGCs, but recent evidence suggests that OFF–center mRGCs draw upon all three types of cones in their RF centers (Field et al., 2010; Klug et al., 2003; Patterson et al., 2019). Incorporating S-cone input into an OFF–center mRGC model is straightforward.

Another question that arises when considering a model of the OFF–center mRGC mosaic is how to split the density of mRGCs in two populations at different eccentricities. In the current model, the density of ON–center mRGCs was assumed to be half of the total mRGC density across all eccentricities. This seems reasonable for central retina where mRGC centers draw primarily on a single cone and where anatomical evidence suggests that each cone provides input to the center of one ON–center and one OFF–center midget bipolar cell. However, there is evidence that the RFs of peripheral ON–center midget (and parasol) RGCs are larger than their OFF–center counterparts in both human and macaque retinas (Kling et al., 2020). This implies that the density of ON–center RGC cells might be lower in the periphery than that of OFF–center cells, given that ON– and OFF–center mRGCs have similar RF overlap (Gauthier et al., 2009). One idea is to treat this asymmetry in mRGC density in an eccentricity-dependent manner, similar to the way we encoded a variable-with-eccentricity RF center overlap.

Finally, when adding an OFF–center mRGC mosaic, one should allow for the possibility of coordination between the ON– and the OFF–center mosaics, to account for recent observations regarding systematic shifts in the spatial layouts of ON– and OFF–center mRGCs (Roy et al., 2021).

### Using the software

The developed software for synthesizing ON–center mRGC mosaics across the retina and for computing with them is part of ISETbio and is freely available at https://github.com/isetbio/isetbio. An introduction to using the mRGCmosaic software is available at:https://github.com/isetbio/isetbio/wiki/Retinal-ganglion-cell-(RGC)-mosaics.

A number of MATLAB tutorials specific to the ON–center mRGCmosaic can be found at https://github.com/isetbio/isetbio/tree/main/tutorials/mrgc. These tutorials demonstrate (a) how to use mosaics of ON–center mRGCs that we have already synthesized at a number of eccentricities, and (b) how to build and validate ON–center mRGC mosaics at any desired eccentricity, using a number of design choices. A summary of current available tutorials is shown in Table 1.

## Figures and Tables

**Fig. 15:**
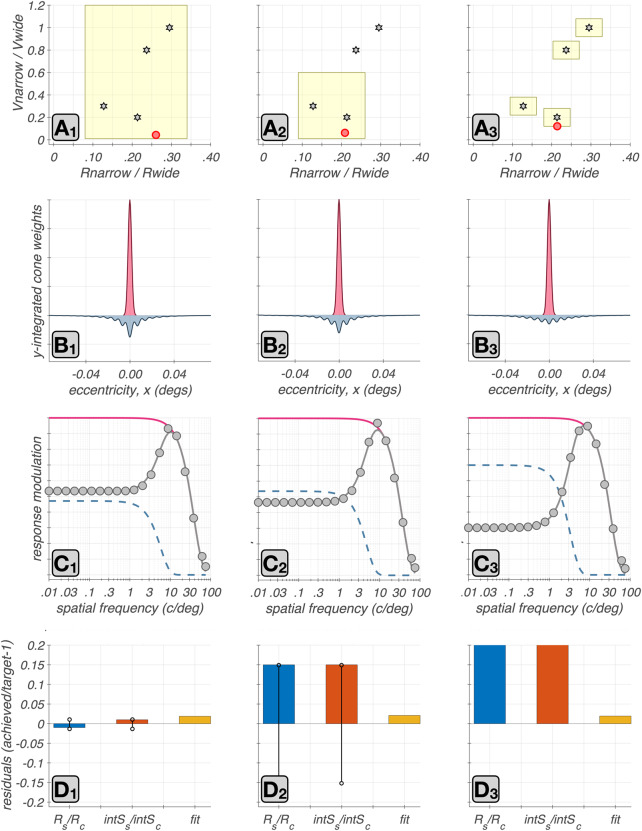
Effect of constraints on surround cone pooling. Results from three options for constraining the surround optimization. Left column: tight tolerance in the parameters of the $\textrm{DoG}$ model fit to the $\textrm{vSTF}$ and loose tolerance in the parameters of the double exponential surround pooling model, $\mathrm W_s(r)$. Middle column: medium tolerance in both sets of parameters. Right column: loose tolerance in the DoG parameters and tight tolerance in the $\mathrm W_s(r)$ parameters. **A1-A3:** The yellow rectangles indicate the tolerance range in the joint space of the two surround cone pooling related parameters, $V_{\textrm{narrow}}/V_{\textrm{wide}}$ and $R_{\textrm{narrow}}/R_{\textrm{wide}}$. Stars depict the corresponding parameter values in four macaque H1 horizontal cells from the study of Packer & Dacey. The red disk depicts the achieved parameter values under each strategy for an example foveal synthetic mRGC. **B1-B3:** Line weighting functions of the retinal space–referred center and surround cone pooling weights under the three examined strategies. **C1-C3:** The $\textrm{vSTF}$ computed under the three strategies (gray disks) and corresponding $\textrm{DOG}$ model fits (gray lines). The red and blue lines depict the center and surround components of the fitted $\textrm{DOG}$ model. **D1-D3:** Blue and orange bars depict the residuals for the ratios of visual space–referred $R_s/R_c$ and $K_s/K_c \times \left( R_s/R_c\right) ^2$ ratios. Black circles connected by a black line depict the enforced tolerance range in these ratios. The enforced tolerance value in D3 was $\tau = 0.5$, and is not visualized. The orange bars depict the $||\textrm{vSTF}(\omega ) - \textrm{DOG}(\omega )||$ residual

## Data Availability

Datasets of synthesized ON-center mRGCmosaics generated during the current study are available at: https://github.com/isetbio/isetbio/tree/main/isettools/ganglioncells/data/prebakedRGCmosaics/ONmRGCmosaics.

## Appendix A: Deriving cone weights to the mRGC RF centers

### A.1 Local topology–based convergent connections (stage 2A)

During the first sub-stage of cone-to-mRGC RF center connectivity, cones are connected to single mRGC RF centers based on the local topology of their respective lattices. Starting with the cell whose RF center is at the most central location of the mRGC lattice, we connect $n_{\mathrm pool}(\epsilon )$ number of L– and M–cones to it, where:

A1 $$\begin{aligned} n_{\mathrm pool}(\epsilon ) = \displaystyle \lfloor \frac{\textrm{D}_{\textrm{cones}}(\epsilon )}{\textrm{D}_{\textrm{mRGC RF}}(\epsilon )} \rfloor \end{aligned}$$

with $\textrm{D}_{\textrm{cones}}(\epsilon )$ and $\textrm{D}_{\textrm{mRGC RF}}(\epsilon )$ being the local spatial densities of the cone mosaic and of the mRGC RF centers, respectively, at the eccentricity, $\epsilon$, of the target mRGC. We draw from the nearest cones that have not yet been connected and whose distance to the mRGC RF center does not exceed a fraction of the local mRGC RF center spacing. This fraction is a parameter of the model and for the work presented here was set to 0.6.

Continuing with these assignments of cones to mRGC RF centers, we move outward to more peripheral locations in the mRGC mosaic, exclusively connecting cones to mRGC RF centers. Any L– and M–cones that remain unconnected at the end of this sub-stage get connected to their nearest mRGC RF center, so that all cones are connected to one mRGC RF center.

This sub-stage can result in local inhomogeneities in both the number of cones and the type of cones pooled within neighboring mRGC RF centers. These inhomonegeities are smoothed out at stage 2B.

### A.2 Optimizing cone connections to mRGC RF centers (stage 2B)

In the second sub-stage of the cone to mRGC RF center connectivity, convergent connections from multiple cones to single mRGC RF centers are optimized according to a desired balance between spatial homogeneity and spectral purity. This is achieved by reassigning cones between nearby mRGC RF centers, which itself occurs in two steps.

In the first step, we allow cone reassignments to a target mRGC from neighboring mRGCs that have a higher input cone numerosity in their RF centers. In the second step, we allow cone swaps between a target mRGC and its neighbors, independently of their input cone numerosities.

The heuristics followed in the first step are as follows. We begin by targeting mRGCs with a single input cone and continue to target mRGCs with progressively higher input cone numerosity. Within each set of targeted input cone numerosity, mRGCs are sorted based on ascending retinal eccentricity. For each targeted mRGC we determine up to 6 neighboring mRGCs which have input numerosity that exceeds that of the target mRGC by at least 2 cones.

Cone reassignments from the candidate donor mRGCs to the target mRGC are executed in multiple passes. Starting with the neighboring mRGC of the highest input numerosity, we determine the best transfer of a single cone. If there are no eligible donor nearby mRGCs, we move to the next targeted mRGC. If there is a single candidate, we accept it and execute the cone transfer. If there are more than one candidates, for each candidate donor mRGC we compute a cost function, $\textrm{C}$, for reassigning each of its cones to the target mRGC, and pick the transfer that minimizes $\textrm{C}$ across all cones and all candidate donor mRGCs. The cost function is described in more detail below.

Once the optimal cone transfers for each mRGC of the targeted input cone numerosity are executed, we move to the next pass, examining possible transfers from neighboring mRGCs of lower input cone numerosity than before, but still higher than the input cone numerosity of the targeted mRGCs. Once all passes are executed, this process is repeated, now targeting mRGCs with increasing input cone numerosity, until all input cone numerosities have been targeted.

In the second step, we only allow for cone swaps between an mRGC RF center and one of its neighbors. For each mRGC of the targeted input cone numerosity, we determine its 6 closest neighbors, but now without regard to their input cone numerosity. For each of these neighboring mRGCs, we evaluate the cost function, $\textrm{C}$, for all possible combinations of cones from the target mRGC and cones from the neighboring mRGC and pick the combination that minimizes $\textrm{C}$. The selected cone swap is executed only if the post-swap value of $\textrm{C}$ is lower than its pre-swap value. Multiple passes through the entire mRGC mosaic are executed, with each pass targeting mRGCs with progressively higher input cone numerosity.

The cost function, $\textrm{C}$, employed to determine the optimal transfer/swap is based on the position and types of the cones pooled by the target mRGC, *t*, and the examined neighboring mRGC, $t_i$. For each examined pair of mRGCs, $(t,t_i)$, $\textrm{C}^{(t,t_i)}$ is defined as:

A2 $$\begin{aligned} \textrm{C}^{(t,t_i)} = \displaystyle \phi \cdot \textrm{C}^{(t,t_i)}_{\chi } + (1-\phi ) \cdot \textrm{C}^{t, t_i}_{\lambda } \end{aligned}$$

where $\textrm{C}^{(t,t_i)}_{\chi }$ quantifies the degree of spatial incompactness, and $\textrm{C}^{(t,t_i)}_{\lambda }$ quantifies the degree of spectral impurity. The $\phi$ parameter controls the desired trade-off between these two RF center attributes. When $\phi = 1$, cone reassignments/swaps are selected so as to minimize the degree of spatial incompactness, when $\phi = 0$, cone reassignments are chosen so as to minimize the degree of spectral impurity, and for intermediate values of $\phi$, cone reassignments are chosen so as to minimize a weighted sum of the two.

The degree of spatial incompactness, $\textrm{C}^{(t,t_i)}_{\chi }$, in Eq. A2 is defined as:

A3 $$\begin{aligned} \textrm{C}^{(t,t_i)}_{\chi } = \textrm{C}^{(t, t_i)}_{\chi _{N}} + \textrm{C}^{(t, t_i)}_{\chi _{o}} \end{aligned}$$

The $\textrm{C}^{(t, t_i)}_{\chi _{N}}$ term quantifies the differential input cone numerosity between the examined pair of mRGCs, and is defined as:

A4 $$\begin{aligned} \textrm{C}^{(t, t_i)}_{\chi _{N}} = \displaystyle \left| \left( N^t_L + N^t_M \right) - \left( N^{t_i}_L + N^{t_i}_M \right) \right| \end{aligned}$$

with $N^t_L$ and $N^t_M$ being the numbers of L– and M–cones pooled by the RF center of mRGC *t*, respectively. The $\textrm{C}^{(t, t_i)}_{\chi _{o}}$ term is a measure of the spatial overlap of the two sets of cones pooled by the two mRGCs, and is defined as the inverse of the distance between the centroids, $(P^t, P^{t_i})$, of the sets of pooled cones normalized by the sum of their respective spatial standard deviations, $(\sigma ^t, \sigma ^{t_i})$:

A5 $$\begin{aligned} \textrm{C}^{(t, t_i)}_{\chi _{o} } = \displaystyle 1 / \left( \frac{||P^t-P^{t_i}||}{\sigma ^t+\sigma ^{t_i}} \right) \end{aligned}$$

A low value of $\textrm{C}^{(t, t_i)}_{\chi _{o}}$ indicates low overlap between the sets of cones pooled by the examined pair of mRGCs and conversely, a high value indicates a large overlap.

The degree of spectral impurity, $\textrm{C}^{t,t_i}_{\lambda }$, in Eq. A2, is defined as the sum of spectral impurities of the RF centers of the pair of analyzed mRGCs:

A6 $$\begin{aligned} \textrm{C}^{t,t_i}_{\lambda } = \displaystyle \textrm{C}^t_{\lambda } + \textrm{C}^{t_i}_{\lambda } \end{aligned}$$

with $\textrm{C}^t_{\lambda }$, quantifying the degree of non-specificity, with regard to the type of cone, in the pooling within the RF center of an mRGC, defined as:

A7 $$\begin{aligned} \textrm{C}^t_{\lambda } = \displaystyle \frac{\min \left( \left[ N^t_L, N^t_M \right] \right) }{N^t_L + N^t_M} \end{aligned}$$

Values of $\textrm{C}^t_{\lambda }$ near zero indicate a low amount of mixture of L– and M–cones, and therefore a RF with a high degree of spectral purity, and conversely, values of $\textrm{C}^t_{\lambda }$, near 0.5, indicate an equal mixture of L– and M–cones, and therefore a RF center with a low degree of spectral purity.

### A.3 Divergent cone connections to multiple mRGC RF centers (stage 2C)

In the final sub-stage of establishing the RF center connectivity, the exclusivity of connections is relaxed, and cone connections are allowed to diverge to more than one mRGC RF center. This divergence is guided by *in-vitro* measurements of mRGC RF center overlap in the macaque (Gauthier et al., 2009).

According to these observations, neighboring mRGC RF centers abut at approximately one standard deviation of their Gaussian RF profile. One caveat of using these *in-vitro* measurements to establish cone divergence in the model, is that these measurements are only available in the far periphery (30–40 degrees), with no data available for more central locations. Anatomical studies suggest, however, that, in the central retina, there must be little to no divergence of cone signals to mRGCs RF centers, so we chose to implement an eccentricity-varying divergence in our model to bridge this gap.

To achieve this, we begin by fitting an ellipsoid to the spatial pooling map of cones that are exclusively connected to the RF center of an mRGC, and extract the rotation, $\alpha$, and the major/minor radii, $\sigma _x, \sigma _y$ of the fitted ellipsoid. Next, a supra-Gaussian ellipsoid function, $G(\textrm{x,y,n})$, defined as:

A8 $$\begin{aligned} \textrm{G}(\textrm{x,y,n}) = \exp { \left[ -0.5 \times \left( \sqrt{({\mathrm {y'}}^2 + {\mathrm {y'}}^2} \right) ^ n \right] } \end{aligned}$$

with:

A9 $$\begin{aligned} \displaystyle \left[ \begin{array}{c c} \mathrm {x'} & \mathrm {y'}\\ \end{array} \right] = \displaystyle \left[ \begin{array}{c c} \textrm{x} & \textrm{y}\\ \end{array} \right] \cdot \left[ \begin{array}{r r} \cos (\alpha ) & -\sin (\alpha ) \\ \sin (\alpha ) & \cos (\alpha ) \end{array} \right] \cdot \left[ \begin{array}{c c} 1/\sigma _x & 0 \\ 0 & 1/\sigma _y \end{array} \right] \end{aligned}$$

is computed by scaling the values of $\sigma _x, \sigma _y$ by a common factor, so that the value of $\textrm{G}(\textrm{x,y,n})$, evaluated at the most remote exclusively-connected cone(s) is $k \times e^{-1/2}$. The value of *k* is determined empirically so that RF maps of nearby mRGCs computed under diffraction-limited optics abut when their sensitivities drop to $e^{-1/2}$ (per Gauthier et al., 2009).

By varying the exponent of the supra-Gaussian, *n*, we model varying degrees of cone divergence. When $n = 10$, we obtain a flat-top Gaussian with very sharp fall-offs, modeling minimal cone divergence. When $n = 2$, we get a standard Gaussian, modeling cone divergence that is consistent with the *in-vitro* measurements of RF center overlap at peripheral locations.

By allowing *n* to vary with eccentricity using a sigmoidal function we obtain a gradual transition in cone divergence with eccentricity. The slope and mid- point of the sigmoidal variation of *n* are currently chosen arbitrarily, with the only restrictions that above 15$^o$, *n* is stable at 2.0, and below 7$^o$, *n* is stable at 10.0. The weights of divergent cone-mRGC RF center connections are computed by evaluating the supra-Gaussian ellipsoid at the positions of all cones in the vicinity of the examined mRGC.

## Appendix B: Deriving cone weights to the mRGC RF surrounds

### B.1 Choosing physiology-based constraints for deriving surround cone weights in stage 3B

The optimization of the parameters of the surround cone pooling functions at each iteration is driven by the residual between the visual STF that is computed based on the surround pooling weights at the previous iteration and the Difference of Gaussians model fit to it, $\textrm{DoG}(\omega )$, which is given by:

B10 $$\begin{aligned} \textrm{DoG}(\omega ) = K_c \cdot R_c^2 \cdot \exp { [-\pi \cdot R_c \cdot \omega ]^2 } - K_s \cdot R_s^2 \cdot \exp { [-\pi \cdot R_s \cdot \omega ]^2 } \end{aligned}$$

This aspect of the optimization captures the observation that the DoG model provides a reasonable fit to the *in-vivo* measured STFs of macaque mRGCs. To ensure adherence to the *in-vivo* data of Croner & Kaplan, the $\textrm{DoG}$ model fit is constrained so that the ratio of surround to center radii, $R_s/R_c$, and the ratio of surround to center integrated sensitivities, $K_s/K_c \times \left( R_s/R_c\right) ^2$, both remain within a specified tolerance, $\tau$, from the corresponding macaque data.

Specifically, for the model’s $R_s/R_c$ ratio, we enforce

B11 $$\begin{aligned} \displaystyle \frac{R^{m}_s}{R^{m}_c} \times \left( 1 - \tau \right) \le \frac{R_s}{R_c} \le \left( 1 + \tau \right) \times \frac{R^{m}_s}{R^{m}_c} \end{aligned}$$

where $R^{m}_c$ and $R^{m}_s$ are the mean values of center and surround radii across the Croner & Kaplan population of macaque mRGCs at the eccentricity of the synthesized mRGC. The model’s $K_s/K_c \times \left( R_s/R_c\right) ^2$ ratio is constrained in the same way.

The residual between the visual STF and the Difference of Gaussians model fit to it, drives the optimization of the surround pooling function. This function is a double exponent (following the H1 horizontal cell spatial RF in the macaque Packer & Dacey, 2002):

B12 $$\begin{aligned} \mathrm W_s(r) = K_{\textrm{wide}} \times \exp \left[ -r/R_{\textrm{wide}} \right] + K_{\textrm{narrow}} \times \exp \left[ -r/R_{\textrm{narrow}} \right] \end{aligned}$$

To ensure that the surround pooling function remains consistent with the parameter values observed in macaque H1 cells (Packer & Dacey, 2002), the optimization of $\mathrm W_s(r)$ is also constrained so that ratio of radii, $R_{\textrm{narrow}}/R_{\textrm{wide}}$, and the ratio of volumes, $V_{\textrm{narrow}}/V_{\textrm{wide}} = K_{\textrm{narrow}}/K_{\textrm{wide}} \times (R_{\textrm{narrow}}/R_{\textrm{wide}})^2$, of the two exponentials both remain within a specified tolerance range of the macaque data.

In the present work, the tolerance range for $R_{\textrm{narrow}}/R_{\textrm{wide}}$ was set to $\left[ 0.07, 0.35 \right]$ for all mosaics, whereas the tolerance range for $V_{\textrm{narrow}}/V_{\textrm{wide}}$ was set to $\left[ 0.01, 0.6 \right]$ for mosaics at eccentricities $\le 15^{\circ }$, to $\left[ 0.3, 0.9 \right]$ for eccentricities in $15^{\circ } \dots 25^{\circ }$, and to $\left[ 0.6, 1.3 \right]$, for eccentricities $\ge 25^{\circ }$.

The joint manipulation of the tolerance values applied to the parameters of the $\textrm{DoG}$ model fit to the $\textrm{vSTF}$, and to the parameters of the double exponential surround pooling model, $\mathrm W_s(r)$, allows for different options for deriving spatial pooling functions in synthetic mRGC surrounds.

One option is to set very strict tolerances on the parameters of DoG model fit while allowing for a large tolerance in the parameters of $\mathrm W_s(r)$. Results of this choice are depicted in the left-most column of Fig. 15. A second option would be to allow medium tolerance levels in both the DoG model fit and the $\textrm{W}_s(r)$. Results of this choice are depicted in the middle column of Fig. 15. A third option would be to enforce strict tolerances in $\mathrm W_s(r)$, for example matching parameters of individual H1 horizontal cells, while allowing for a loose tolerance in the DoG model fit. Results of this choice are depicted in the right column of Fig. 15. In the present work, we chose the second option.

## Code Availability

The code used to generate the data, and various tutorials on how to use the software are available at: https://github.com/isetbio/isetbio/tree/main. An introduction to using the software is available at: https://github.com/isetbio/isetbio/wiki/Retinal-ganglion-cell-(RGC)-mosaics.
